# Structure and Properties of Al–CNT-Based Composites Manufactured by Different Methods: A Brief Review

**DOI:** 10.3390/ma18010214

**Published:** 2025-01-06

**Authors:** Marat Nurguzhin, Marat Janikeyev, Myrzakhan Omarbayev, Azira Yermakhanova, Mohammed Meiirbekov, Miras Zhumakhanov, Aruzhan Keneshbekova, Meiram Atamanov, Aigerim Akylbayeva, Aidos Lesbayev, Darkhan Yerezhep

**Affiliations:** 1 JSC “National Center for Space Research and Technology”, Shevchenko Str., 15, Almaty 050010, Kazakhstan; nurguzhin.m@spaceres.kz (M.N.); m.janikeyev@spaceres.kz (M.J.); omarbayev.m@spaceres.kz (M.O.); a.yermakhanova@spaceres.kz (A.Y.); meiirbekov.m@spaceres.kz (M.M.); zhumakhan.miras@spaceres.kz (M.Z.); 2 International Chinese-Belorussian Scientiffc Laboratory on Vacuum Plasma Technology, Nanjing University of Science and Technology, 200 Xiaolingwei Str., Nanjing 210094, China; 3 Institute of Natural Science, Kazakh National Women’s Teacher Training University, Almaty 050000, Kazakhstan; mk.atamanov@gmail.com; 4 Institute of Energy and Mechanical Engineering, Satbayev University, 22a Satpaev Str., Almaty 050013, Kazakhstan; a.akylbayeva@satbayev.university (A.A.); a.lesbayev@satbayev.university (A.L.)

**Keywords:** Al-CNT, composites, Al_4_C_3_ phases, interfacial bonding, microstructure, powder metallurgy

## Abstract

Aluminum–carbon nanotube (Al–CNT) composites represent a cutting-edge class of materials characterized by their exceptional mechanical, thermal, and electrical properties, making them highly promising for aerospace, automotive, electronics, and energy applications. This review systematically examines the impact of various fabrication methods, including conventional powder metallurgy, diffusion and reaction coupling, as well as adhesive and reaction bonding on the microstructure and performance of Al–CNT composites. The analysis emphasizes the critical role of CNT dispersion, interfacial bonding, and the formation of reinforcing phases, such as Al_4_C_3_ and Al_2_O_3_, in determining the mechanical strength, wear resistance, corrosion resistance, and thermal stability of these materials. The challenges of CNT agglomeration, high production costs, and difficulties in controlling interfacial interactions are highlighted alongside potential solutions, such as surface modifications and reinforcement strategies. The insights presented aim to guide future research and innovation in this rapidly evolving field.

## 1. Introduction

Modern technologies are imposing increasingly stringent requirements on structural materials, which must combine high strength, minimal weight, and sufficient ductility. Achieving this balance is a significant challenge for many industries, including the aerospace and automotive ones. Materials need to resist corrosion, endure extreme temperatures and aggressive environments, exhibit high wear resistance, maintain stable electrical and magnetic properties, and remain cost-effective [1,2,3]. To meet these demands, composite materials are developed, combining two or more components with different physical, chemical, and mechanical properties. Such materials are formed by dispersing one or more reinforcing phases within a continuous matrix. Depending on the matrix type, composites can be polymer-based (PMC) [4], ceramic-based (CMC) [5], or metal-based (MMC) [6].

Aluminum and its alloys, known for their high strength, low density, and manufacturability, are among the most sought-after lightweight structural materials [7,8]. However, industries such as the automotive and aerospace ones are actively working to reduce structural weight to improve fuel efficiency and decrease harmful emissions that negatively impact the climate and environment [9,10].

A notable example of advanced material applications is the Boeing 787 Dreamliner, marking a new era in using composites and innovative metals to enhance aircraft efficiency and performance [11]. The Boeing 787 utilizes titanium and aluminum alloys extensively, accounting for approximately 14% and 20% of the structural weight, respectively. Meanwhile, composites constitute around 50% of the structure [12], compared to only 12% in its predecessor, the Boeing 777 [13].

Today, aluminum-based metal matrix composites are among the most widely used and promising materials, combining the strength of metals with the enhanced properties provided by various reinforcements. For instance, ceramic particles (Al_2_O_3_, SiC, TiC, B_4_C, etc.) improve wear resistance and hardness [14,15,16,17,18], but their use is limited by processing difficulties, the formation of brittle phases (e.g., Al_4_C_3_), and weak interfacial interactions.

On the other hand, over the past two decades, carbon nanomaterials (graphene sheets, carbon nanofibers, and carbon nanotubes) have been actively used as reinforcements in composite manufacturing [19,20,21,22]. Among these, carbon nanotubes (CNTs) stand out due to their exceptional properties, including high specific strength (up to 55.55 GPa·mg^−1^·m^−3^), a Young’s modulus of about 1 TPa, tensile strength up to 100 GPa, low thermal expansion coefficient, and excellent thermal conductivity [23,24,25]. Additionally, CNTs exhibit a high aspect ratio, excellent chemical stability, and outstanding electrical properties [26]. Combining an aluminum matrix with CNTs enables the production of composites that retain the lightweight properties of aluminum while offering improved mechanical, thermal, and electrical performance due to CNT reinforcement. These materials hold great promise for applications in aerospace, transportation [27], electronics [28], energy [29], and environmental systems [30].

**Figure 1:**
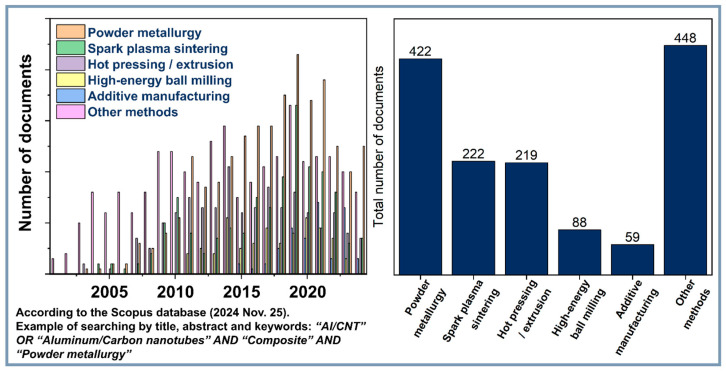
Number of documents published in the Scopus database from 2000 to 2025.

The growing number of publications on this topic (Figure 1) reflects the increasing interest of the scientific community, particularly in developing new synthesis methods and studying the properties of Al-CNT composites. Since the pioneering work by Kuzumaki et al. [31] on hot pressing and extrusion in 1998, significant progress has been made. However, unresolved challenges remain, such as the high degree of CNT agglomeration due to strong van der Waals interactions and weak interfacial bonding with the matrix (poor wettability). These factors reduce the reinforcing effect of nanotubes and limit the composite’s applications.

Currently, extensive research is being conducted on the effects of CNTs on various matrices. Researchers are addressing the dispersion challenge using advanced powder metallurgy methods [32,33,34,35,36,37], such as hot isostatic pressing, spark plasma sintering (SPS), and rapid solidification. Despite these efforts, the industrial implementation of Al-CNT composites faces significant challenges, including poor weldability [38] and equipment limitations for producing large-scale components [39].

To improve weldability, solid-state bonding (SSB) technologies such as diffusion welding [40], hot pressing [41], rolling [42], and additive forging [43] have been proposed. Research shows that controlling the bonding temperature (BT) enhances weld seam strength and prevents structural softening [44].

This review aims to systematize existing approaches to fabricating Al-CNT composites, analyze their morphological and structural characteristics, and investigate the relationship between fabrication methods and material properties. It discusses modern synthesis methods, CNT dispersion challenges, interfacial interaction mechanisms, and optimization strategies. The authors hope this review will significantly contribute to identifying promising research directions and addressing current challenges for the industrial application of Al-CNT composites.

## 2. Fabrication Methods of Al-CNT Composites and Their Impact on Structure

**Figure 2:**
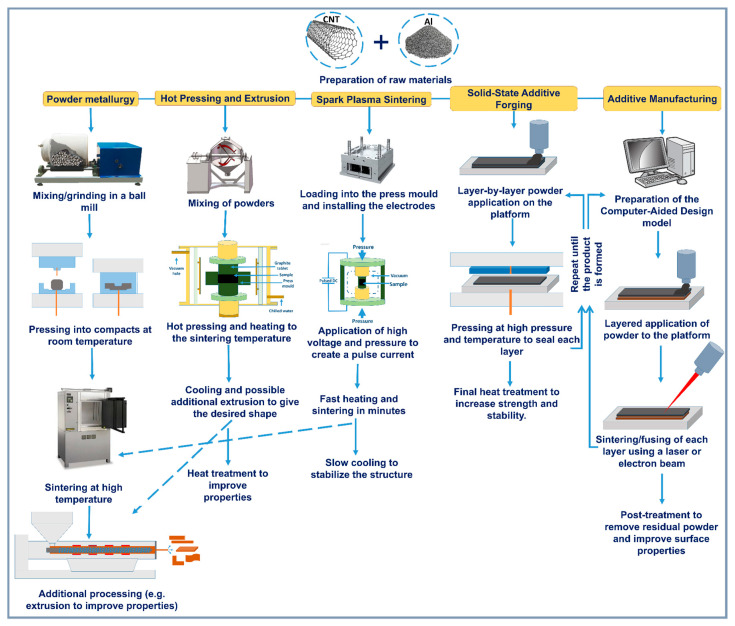
Schematic diagram of Al-CNT synthesis using various methods.

The fabrication process of Al-CNT composites plays an important role in the formation of their microstructure and, as a consequence, determines their mechanical, thermal and physicochemical properties. Successful incorporation of CNTs into the aluminum matrix requires efficient processing that ensures uniform distribution of CNTs, minimizes their agglomeration and prevents nanotube damage during synthesis. Depending on the chosen synthesis method, different interactions between the matrix and CNTs occur, which affects the formation of interfacial junctions, grain size and the presence of defects. Some of the main methods for producing aluminum composites are shown in schematic Figure 2.

To achieve optimal composite performance, processing conditions such as temperature, pressure, dwell time and cooling rate must be carefully controlled. Methods based on mechanical bonding, such as powder metallurgy and high-energy ball milling (HEBM), provide well-dispersed CNTs and allow for the control of the material microstructure [45,46]. At the same time, diffusion and reaction bonding techniques, including spark plasma sintering (SPS) and hot pressing or extrusion (HP or HE), provide dense compaction and strong interfacial bonds [47,48,49], but require control to prevent the formation of undesirable phases such as Al_4_C_3_. Liquid-phase methods, such as injection molding and melt infiltration, produce dense composites, but carry the risk of CNT agglomeration and reactive interaction with the aluminum matrix at high temperatures [50,51]. Coating techniques, such as chemical and physical vapor deposition, provide thin reinforcement layers, improving adhesion between CNTs and the matrix [52], although it can be difficult to produce on a large scale. Each synthesis method has its own limitations and opportunities. Some technologies are only applicable in the laboratory or for specific applications, while others require optimization for industrial production.

In addition, the inclusion of various alloying additives can significantly improve the mechanical and physicochemical characteristics of the composite [53,54]. For example, the addition of magnesium [55] and copper [56] increases hardness and strength, while oxides and nonmetals enhance resistance to aggressive conditions. Some additives also help to reduce the agglomeration of CNTs [57,58] by ensuring their uniform distribution and improving the adhesion between the matrix and nanotubes.

Thus, the choice of processing technology plays a key role in achieving Al-CNT composites with improved properties and minimal defects. This chapter discusses the main methods of obtaining such composites by the type of interaction between matrix and CNTs, their influence on the structure and approaches to optimize the processes to meet the existing technological challenges.

### 2.1. Conventional Powder Metallurgy Techniques

**Figure 3:**
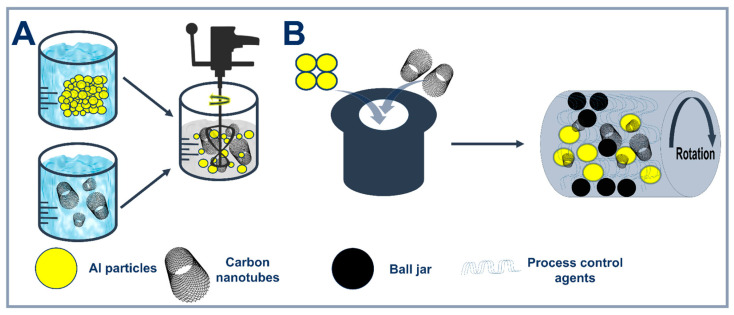
Schematic representation of wet mixing (**A**) and ball milling (**B**) used to prepare Al–CNT composites.

Methods for producing Al-CNT composites based on mechanical joining are an effective approach to integrate reinforcing components into a metal matrix by solid-phase processing. The development of methods based on mechanical bonding comes from the advent of technologies that enabled composites’ preparation by mixing powders and reinforcing particles at relatively low temperatures. Powder metallurgy (PM) was one of the first and most effective approaches to achieve a uniform distribution of reinforcing particles in the matrix through careful mixing of powders and their subsequent sintering. One of the key steps in PM is powder preparation, which includes wet mixing (ultrasonic or mechanical mixing) and ball milling, often used in combination (Figure 3). HEBM is an advanced ball milling method capable of operating at higher intensities, which allows researchers to achieve both the dispersion of CNTs at the microscopic level and to change the structure of the matrix particles, improving their mechanical properties. Despite the advantages, this method has a number of limitations associated with the risk of formation of various interfaces and undesirable reactions between components. In addition, the surface morphology of CNTs may be changed under intensive mechanical impact on CNTs, which may affect the composite properties.

Further development of mechanical synthesis methods is due to the application of such technologies as friction stirring bonding (FSP). This relatively new process allows for the modification of the surface layers of composites, improving their microstructure and mechanical properties due to intensive plastic deformation and dynamic recrystallization.

Though there is an abundance of different technologies for the production of aluminum and CNT-based MMCs, these methods face many limitations related to the processing of the composites. The most important of these are the chemical and structural stability of CNTs, uniform dispersion of CNTs, and the quality of bonding at the interface between CNTs and matrix. Further in this paper, the main mechanical joining technologies, their advantages, limitations and influence on the properties of composites will be discussed in detail.

#### 2.1.1. Powder Metallurgy (PM)

Powder metallurgy (PM) is one of the oldest and most widespread methods for the production of composite materials, which continues to evolve due to its energy efficiency and ability to control the microstructure of materials, including the size, shape and distribution of reinforcing particles in the matrix [59]. The implementation of PM for the synthesis of composites based on metal particles and carbon nanotubes (Me-CNT) is due to its simplicity (Figure 3) and functionality [60].

**Figure 4:**
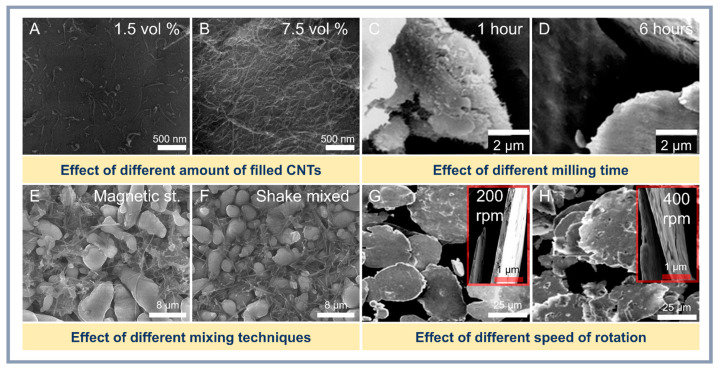
Influence of CNT content (**A**,**B**) [64], milling time (**C**,**D**) [65], mixing method (**E**,**F**) [66], and rotation speed (**G**,**H**) [67] on the morphology and structure of Al-CNT composites.

However, the method requires careful optimization of parameters to minimize undesirable effects and maximize material performance. One of the key steps of PM is the mechanical action on the starting powders, such as ball milling, which promotes uniform dispersion of CNTs in the aluminum matrix. For example, Fan et al. [61] showed that using the flake PM method can achieve a homogeneous distribution of not only CNTs but also other nanoscale particles such as Al_2_O_3_ and B_4_C. This is achieved by reducing agglomeration and improving interfacial interaction. It should be taken into account that grinding time and rotational speed during ball milling play an important role in the structure formation of composites. For instance, increasing the grinding time favors the destruction of CNT agglomerates and an improvement in their distribution (Figure 4B). However, excessive grinding can damage nanotubes, reducing their reinforcing capacity [62]. High rotational speeds also improve adhesion at the interfacial boundary due to more intensive mixing, but are accompanied by the risk of local overheating and agglomeration of aluminum particles [58].

The strength of composites is usually proportional to the volume fraction of reinforcement as stated in the mixing rule [63]. However, this dependence is not observed for Al-CNT composites. Studies show that the hardening efficiency decreases with increasing CNT content: at concentrations above 2 wt.%, hardening can become negative [58]. This is due to the agglomeration of CNTs, which disrupts the uniform distribution in the matrix, causes structural defects and hinders the diffusion of aluminum atoms, resulting in porosity and reduced composite density. On the other hand, with prolonged sintering, higher density values can be achieved, but, at the same time, grain enlargement can occur, which leads to a reduction in the number of grain boundaries and facilitates the movement of dislocations.

Hence, it can be noted that the sintering process plays a key role in the formation of the composite microstructure. By optimizing parameters such as temperature, residence time and pressure, uniform particle bonding and density increase can be achieved. For example, increasing the sintering temperature from 400 to 600 °C increases the microhardness by 19.1% and the modulus of elasticity by 20.2% In an analogous work, Kim et al. [68] showed that annealing at a temperature of 873 K and a pressure of 600 MPa allows for the achievement of a density of 98.9% and a hardness of 1.4 GPa. However, exceeding the optimum values causes the formation of brittle Al_4_C_3_ phase, which reduces the ductility of composites [69]. In order to minimize this effect, Toozandehjani et al. [70] proposed decorating CNTs with aluminum oxide, which improves the interfacial interaction and increases the strength of the composite. Another study [71] reported that the introduction of Si suppresses the formation of Al_4_C_3_ and improves the distribution of CNTs, improving the strength and durability of the composite.

**Figure 5:**
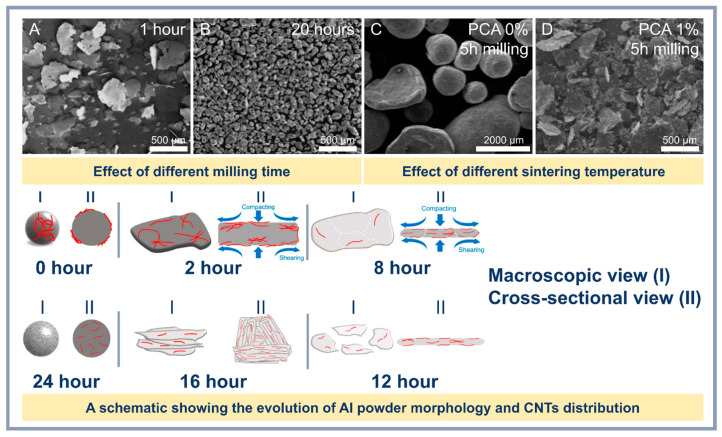
The influence of milling time (**A**,**B**) [73] and sintering temperature (**C**,**D**) on the morphology and structure of Al–CNT composites [74]. Schematic illustration of morphology changes relative to milling time [46].

HEBM is increasingly being used to improve the distribution of CNTs in the aluminum matrix. Xu et al. [62] demonstrated that the combination of low- and high-speed milling resulted in a composite with a tensile strength of 376 MPa at 1.5 wt.% CNTs. However, excessive milling leads to the incorporation of CNTs into aluminum pellets and deterioration of their distribution, as noted by Wan et al. [72]. As a result of the intense mechanical impact of grinding balls during the grinding process, the morphological features of CNTs may be affected and there is a higher probability of forming metallic bonds between aluminum particles by plastic deformation (cold welding). However, to reduce this effect, process control agents (PCA) are used, which are able to adsorb on the surface of the powders and reduce the surface tension, preventing cold welding. The impact of different synthesis parameters on the structures can be seen in Figure 5.

Other challenges faced by PM include the formation of oxide films on the surface of the powders, which hinders diffusion and reduces the quality of the bond between CNTs and the matrix [75]. However, this layer can be removed by degassing the mixture in an inert atmosphere. Since grinding does not lead to complete melting, the phase distribution is retained in the composite powders.

Currently, powder metallurgy remains one of the most versatile and simple methods for producing Al-CNT composites. However, the development of hybrid methods combining powder metallurgy with other processes such as additive manufacturing or chemical deposition represents a promising area for further growth. For example, the molecular mixing method (MLM) can achieve a homogeneous distribution of CNTs at the molecular level, which significantly improves the interfacial interaction and mechanical properties of the composites [76].

#### 2.1.2. Friction Stirring Bonding (FSP)

In recent years, great attention has been paid to the production of metal matrix composites by frictional stirring processing (FSP). This method allows for the formation of an ultrafine grain structure in the machining zone due to intensive plastic deformation and dynamic recrystallization [77,78]. FSP uses a rotating tool that is embedded in the surface of the processed material and moves along a given direction, creating local heating and deformation, which leads to grain refinement and improved distribution of reinforcing particles. FSP reduces residual stresses compared to other severe plastic deformation methods such as equal-channel angular extrusion and accumulative bundling with rolling [79,80].

The optimization of the FSP parameters is critical to obtain homogeneous composites with improved properties. For instance, Sharma et al. [81] used multiple micro-sized channel reinforcement filling (MCRF) for uniform distribution of CNTs, which resulted in grain refinement to ~7.18 µm and improved wear resistance by 20% due to the formation of a carbon-containing tribolayer. At the same time, the use of a single macro-sized channel reinforcement filling (SCRF) resulted in less uniform CNT distribution and worse strengthening. Increasing the rotational speed and number of passes promotes better mixing and distribution of reinforcing particles. In the research of Kumar et al. [82], three-pass processing at 1600 rpm and 20 mm/min resulted in uniform distribution of SiC and CNT and reduction in grain size to 5.24–6.36 μm due to dynamic recrystallization (CDRX, DDRX). However, excessive rotational speed can cause the degradation of reinforcing particles as shown in [83], where the MWCNT structure was destroyed by high temperature and deformation.

FSP leads to grain refinement and changes in the morphology of the composites. The authors of [84] showed that the parameters of 270 rpm and 78 mm/min provide a uniform SiC distribution and reduce the grain size to 5.45 μm, which increases the microhardness of AA2014 composite. Meanwhile, the agglomeration of reinforcing particles remains one of the major problems. For instance, Khan et al. [85] reported that the addition of B_4_C improved the strength and hardness of the Al-5083 composite, but the non-uniform distribution of MWCNTs reduced the ductility by 50%. The solution to the agglomeration problem may be to optimize the method of introducing the reinforcing component. Pragada et al. [86] reported that changing the direction of tool movement after each pass improved the uniformity of SiC and CNT distribution and increased the microhardness to 133.2 HV. In another study [87], an artificial neural network (ANN) was used to model the effect of the matrix/reinforcement particle size ratio (Rs/Ms) on wear resistance and to identify the optimum parameters to minimize wear.

Zhang et al. [88] reported that using parameters of 475 rpm and 60 mm/min, interfaces between CNT and Al were formed, resulting in a 50% increase in strength without loss of electrical conductivity. Furthermore, the combination of FSP and rolling at 753 K favored a uniform distribution of CNTs and the formation of a strong interface, resulting in a tensile strength of 600 MPa at 10% elongation [89]. Nevertheless, the formation of undesirable phases at the interface remains a challenge. The authors of [83,90] report that high process temperatures led to the formation of aluminum carbide (Al_4_C_3_), which negatively affected the reinforcement properties of CNTs. A possible solution is to use optimal temperature conditions and multi-pass processing to minimize the formation of Al_4_C_3_ and other brittle phases. FSP activates several hardening mechanisms such as load transfer, dislocation pinning and Orowen mechanism. For example, Du et al. [91] reported that the addition of Al_2_O_3_ and CNT resulted in grain refinement to 3.17 μm and increased hardness to 108.4 HV due to pinning of grains by nanoparticles. However, an excessive amount of reinforcing particles can cause cracking and reduced ductility [87].

Kumar et al. [92] established the optimal processing parameters (1600 rpm and 30 mm/min) at improved wear resistance of the AA7075-B_4_C composite due to uniform particle distribution and improved surface integrity. However, one of the major problems is the degradation of the carbon nanotube (MWCNT) structure at high temperatures occurring during FSP [93]. The degradation of the MWCNT tubular structure led to the formation of polyaromatic and turbostratic carbon structures, which reduced the reinforcement efficiency [83].

Nevertheless, studies demonstrate that friction stirring is an effective method for creating metal composites with improved mechanical and tribological properties. However, to achieve optimal results, the problems of agglomeration, degradation of reinforcing components and brittle phase formation must be taken into account. Solving these problems requires careful optimization of the process parameters, surface modification of reinforcing particles and a combination of different processing methods. Prospects for further research include the development of new hybrid composites and the use of modeling to predict optimal processing conditions.

### 2.2. Diffusion and Reaction Coupling

Diffusion and reaction coupling methods are based on heat treatment at high temperatures and pressures, which promotes the diffusion of atoms between the matrix and reinforcing particles, as well as the formation of new phases at the interface. This approach allows researchers to achieve uniform distribution of carbon nanotubes and minimize porosity, which is critical for improving the mechanical and physicochemical characteristics of composites. Diffusion coupling provides a strong bond between the components due to the movement of atoms across the interfaces under the action of temperature and pressure.

However, high processing temperatures can lead to the formation of brittle phases such as Al_4_C_3_, which negatively affects the ductility and strength of the composites. This section reviews the main diffusion and reaction coupling technologies, their impact on the structure and properties of Al-CNT composites, and possible ways of optimization to address the current technological challenges.

#### 2.2.1. Spark Plasma Sintering (SPS)

Spark plasma sintering (SPS) is an advanced powder metallurgy method that allows for a significant reduction in processing time and efficient preservation of CNT properties [94,95,96]. SPS employs a pulsed electric current and applied pressure to rapidly and uniformly sinter composites at relatively low temperatures. This process favors the formation of a dense structure with minimal porosity and uniform CNT distribution [97]. In comparison with conventional powder metallurgy, SPS has been shown to assist in the prevention of excessive formation of aluminum oxides, thereby achieving a higher level of microstructural control [98].

**Figure 6:**
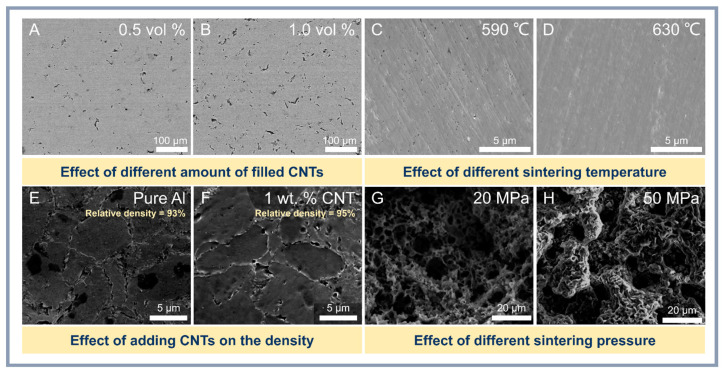
The influence of CNT content (**A**,**B**) [99], sintering temperature (**C**,**D**) [105], CNT addition (**E**,**F**) [106], and sintering pressure (**G**,**H**) [107] on the morphology and structure of Al-CNT composites.

Optimization of the SPS parameters such as temperature, pressure and curing time is critical to obtain composites with improved performance (Figure 6). For example, sintering at 550–575 °C and 37 MPa achieves a uniform distribution of CNTs and minimizes agglomeration, resulting in hardness increases of up to 82 HV and tensile strengths of up to 250 MPa [97]. At the same time, increasing the temperature up to 600 °C promotes the formation of aluminum carbide (Al_4_C_3_), which can negatively affect the ductility [99]. According to Suslova et al. [100], increasing the sintering temperature and pressure improves the composite’s density and reduces the number of CNT defects, but promotes the formation of mesopores. In a study by Wan et al. [101], varying the parameters of SPS and subsequent hot extrusion (HE) at 600 °C produced heterostructured composites with tensile strength up to 549 MPa and elongation up to 11.2%.

The addition of CNTs and their modification play a key role in determining the final characteristics of composites. For example, in [76], the addition of 0.5 wt.% CNTs to NiAl matrix led to an increase in compressive strength to 831 MPa and yield strength to 429 MPa due to grain pinning and bridging crack formation. However, exceeding the optimum CNT content led to the agglomeration and deterioration of properties due to increased porosity [99,102]. Hybrid reinforcement, such as a combination of CNT and SiCp particles, provides a synergistic effect. Al6061 composites reinforced with 2 vol% CNT and SiCp showed a 37% increase in hardness and 38% increase in tensile strength compared to pure aluminum composites [103]. In [104], the use of CNTs modified with a titanium coating (CNTs@Ti) improved tensile strength to 284 MPa at 10% elongation.

One of the key challenges in using SPS is controlling the formation of interfacial compounds such as Al_4_C_3_, which can reduce the ductility of composites. The authors of [99] showed that sintering at 500–550 °C minimized the formation of Al_4_C_3_, improving the hardness of the composite to 122 HV and compressive strength to 404 MPa. Meanwhile, the use of oxide films formed during the pre-treatment of powders can hinder the reactions of CNTs with the aluminum matrix and contribute to the formation of Al-CNT_2_O_3_-type structures [72,95].

SPS contributes to grain refinement and improved mechanical properties through dynamic recrystallization and pinning of dislocations with reinforcing particles. An increase in tensile strength to 268 MPa was achieved in Al composites reinforced with hybrid CNT and RGO with an average grain size of 244.3 nm [108]. Similarly, in TiAl composites sintered at 1000 °C and 30 MPa, a content of 0.4 wt.% CNT resulted in a reduction in the average grain size to 32 microns and an increase in hardness to 377 HV [102].

Combining SPS with other processing methods, such as HE and hot rolling, further improves the properties of composites. In [97], hot rolling after SPS at 500 °C improved CNT distribution and increased tensile strength to 250 MPa at 8% strain. This demonstrates the importance of sequential processing to achieve optimum microstructure and mechanical performance. SPS is a promising technology for producing composites with improved mechanical properties and controlled microstructure. Successful application of SPS requires careful optimization of process parameters, control of interface formation and modification of reinforcing particles. Hybrid methods and additional processing allow for synergistic effects to be achieved, making SPS in demand in areas such as aerospace, electronics and energy.

#### 2.2.2. Hot Pressing and Extrusion (HP and HE)

**Figure 7:**
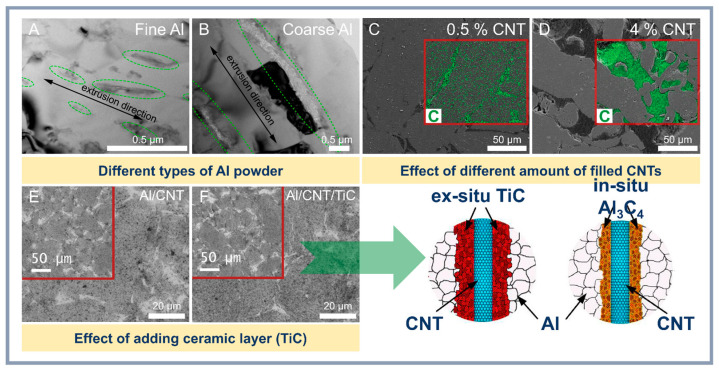
The influence of aluminum type (**A**,**B**) [115], CNT content (**C**,**D**) [116], and the addition of various additives (**E**,**F**) [117] on the morphology and structure of Al-CNT composites.

To improve the density and tensile strength of composites produced by traditional methods, various post-processing techniques are recommended. Hot pressing (HP) and hot extrusion (HE) are effective methods for producing Al-CNT composites with high density, improved microstructure, and optimized mechanical properties. These methods rely on the application of high temperatures and pressures, which help eliminate porosity, enhance diffusion between particles, and form strong interfacial bonds. However, the successful implementation of these processes requires careful optimization of parameters such as temperature, pressure, dwell time, deformation rate, and concentration of CNTs and different additives (Figure 7).

HP allows for the sintering of pre-prepared powders at a temperature close to the melting point of aluminum under applied pressure [109]. This method helps eliminate pores, improve density, and form strong interfacial bonds between the aluminum matrix and carbon nanotubes. Research by Zuo et al. [44] demonstrated that hot pressing at temperatures of 455–515 °C improves the composite’s density and interfacial quality. However, increasing the temperature beyond 515 °C leads to the formation of the brittle Al_4_C_3_ phase, which reduces the material’s ductility. The introduction of alloying additives such as silicon [110] and magnesium [111] helps stabilize the microstructure and minimize undesirable reactions. For example, the addition of Si suppresses the formation of Al_4_C_3_ and enhances the bonding between CNTs and the aluminum matrix [112]. A study by Kim et al. [68] showed that annealing at 600 °C and a pressure of 600 MPa allows researchers to achieve a density of 98.9% and a hardness of 1.4 GPa. The addition of magnesium improves mechanical strength and ductility by stabilizing the interfacial regions [113]. However, despite the advantages, hot pressing is associated with the risk of thermal damage to CNTs at high temperatures, which can lead to a reduction in their reinforcing properties [114]. To minimize these effects, methods for pre-modifying the surface of CNTs, such as decorating the nanotubes with aluminum oxides [70], are used to improve the interfacial interaction and enhance the composite’s strength.

HE is a crucial post-processing step for Al-CNT composites, providing intense plastic deformation and improving the material’s microstructure. The study by Li et al. [118] demonstrated that extrusion at 570 °C minimizes CNT agglomeration and results in composites with high tensile strength. Hot extrusion also enhances ductility and reduces porosity by effectively densifying the structure. For example, Xu et al. [62] achieved a tensile strength of 376 MPa with 1.5 wt.% CNTs by combining ball milling with subsequent hot extrusion. The intense plastic deformation during extrusion promotes grain refinement and improves grain orientation, leading to anisotropy in material properties.

Combining hot pressing and hot extrusion provides a synergistic effect from both processes. Hot pressing ensures preliminary densification and sintering of powders, while extrusion completes composite formation, improving its microstructure and texture. For instance, Xiang et al. [113] used hot pressing at 600 °C followed by extrusion at 500 °C to produce composites with minimal CNT agglomeration and high strength. Studies show that the processing sequence is critical for achieving optimal properties. For example, hot pressing at moderate temperatures followed by extrusion under controlled parameters helps avoid CNT damage and preserves their reinforcing properties [71].

Despite significant progress, hot pressing and extrusion face several challenges. One major issue is the risk of brittle phase formation and thermal degradation of CNTs at high temperatures. Additionally, intense deformation may lead to structural defects and non-uniform CNT distribution. Addressing these issues requires further development of hybrid technologies combining HP and HE with other methods, such as HEBM and additive manufacturing [76].

#### 2.2.3. Die Casting

The die casting method plays a crucial role in the production of bulk composite samples, enabling the achievement of high mechanical properties. However, this method requires careful parameter optimization to minimize defects and improve the microstructure. For example, Oh et al. [119] report that die casting with oxygen replacement reduces porosity and enhances the distribution of carbon nanotubes in composites based on the A383 Al-Si-Cu alloy. An optimal CNT content of 1 wt.% increases the tensile strength to 258.5 MPa and hardness to 157.9 HV, attributed to the formation of Al_2_O_3_ oxide phases and the uniform distribution of nanotubes. However, the authors note that increasing the CNT content to 2 wt.% causes agglomeration and porosity growth, reducing the material’s mechanical properties.

On the other hand, Popov et al. [120] demonstrated that combining die casting with cyclic extrusion improves the mechanical properties of Al1070 alloy-based composites reinforced with 0.5 wt.% MWCNT. After ten extrusion cycles at 330 °C, the tensile strength increases to 132.2 MPa. However, corrosion tests in a 3% NaCl solution showed a reduction in corrosion potential to −745 mV, highlighting the need for optimized corrosion protection. Melt stirring before die casting also facilitates a more uniform distribution of carbon nanotubes in the matrix. Usef et al. [121] found that composites based on the Al-7Si-0.35Mg alloy produced by pre-mechanical alloying exhibit a 44% reduction in average grain size and a 2.5% improvement in thermal conductivity. Additionally, they noted that the formation of aluminum carbides at grain boundaries provides additional strengthening, resulting in a 19.8% increase in yield strength and a 14.13% increase in tensile strength. Zhang et al. [122] point out that heat treatment after die casting is an effective method for enhancing the characteristics of composites. In Al-Si-Mg-Mn-based composites, an aging regime at 225 °C achieves a yield strength of 240 MPa due to the formation of the Mg_2_Si phase and a high density of needle-like β″-phases of Mg_5_Si_6_. The authors also note that the use of numerical models to optimize heat treatment allows for property prediction and microstructure improvement of the composites.

Combining carbon nanotubes with other reinforcing components, such as boron carbide particles, results in synergistic effects. For instance, Maniraj et al. [123] reported that Al6061 composites with 3 wt.% B_4_C and 9 wt.% CNT additions demonstrate a tensile strength of 250 MPa and a hardness of 52.7 BHN. Strengthening is associated with increased interface stiffness and effective dislocation pinning. However, increasing the CNT content beyond the optimal level causes agglomeration, limiting further improvements in mechanical properties.

Despite the progress achieved, Oh et al. [119] and Larianovsky et al. [124] note that CNT agglomeration at high concentrations reduces the mechanical properties of composites. Usef et al. [121] also showed that the formation of Al_4_C_3_ at grain boundaries can both improve strength and negatively affect the material’s ductility. The addition of CNTs may decrease the corrosion resistance of composites, necessitating the development of additional protective methods [96]. Optimization of process parameters, including temperature, pressure, and CNT dispersion methods, is a key factor for achieving high-performance composites [125,126]. Thus, the combination of various processing methods and the use of hybrid reinforcing components offer broad prospects for creating composites with tailored operational properties.

### 2.3. Adhesive and Reactive Bonding

Adhesive and reactive bonding represent a group of methods aimed at creating composite materials by forming thin layers or coatings of reinforcing components on the surface of a metallic matrix. These methods improve interfacial interaction, ensure a uniform distribution of the reinforcing filler, and enable control over the structure at the nano- and microlevels. Unlike mechanical or diffusion bonding methods, adhesive and reactive techniques provide a high degree of control over the composition and thickness of applied coatings, minimizing defect formation and enhancing the mechanical and functional properties of composites.

Vapor deposition methods, such as chemical vapor deposition (CVD) and physical vapor deposition (PVD), enable the creation of uniform and dense coatings on the surface of the metallic matrix. These methods are characterized by high purity and allow for the precise control of the morphology and thickness of the coatings, making them effective in enhancing interfacial adhesion and preventing the agglomeration of CNTs.

Furthermore, laser spraying and electrolytic deposition allow for the application of reinforcing components with high precision and minimal thermal deformation of the matrix, which is particularly important for heat-sensitive materials. Thus, the use of adhesive and reactive bonding opens up broad opportunities for creating aluminum-alloy-based composites with improved properties. These methods provide precise control over the structure and composition of interfacial regions, contributing to enhanced strength, hardness, and corrosion resistance of the resulting materials.

#### Deposition Methods

The CVD method is one of the most effective techniques for depositing CNTs onto metallic matrices and reinforcing particles [127,128]. In the work by Liu et al. [129], CNT deposition onto aluminum powders was carried out at 873 K using nickel nanocatalysts. The results demonstrated that uniform CNT distribution and the formation of Al_4_C_3_ nanostructures enhanced the composite strength to 191 MPa and elongation to 32.6%. Similarly, Tang et al. [130] employed polymer pyrolysis with CVD to deposit CNTs onto aluminum nanoplatelets. An optimal temperature of 600 °C yielded CNTs 420–450 nm in length with high purity, confirmed by a low ID/IG value of 0.64 in Raman spectra.

In the study by Wang et al. [112], CVD was used to deposit CNTs onto SiCp particles, creating hybrid reinforcement in 7075Al-Mg composites. The resulting materials achieved a tensile strength of 650.8 MPa due to the bridging effect of CNTs and the formation of MgZn_2_ phases at the interface. However, it was noted that CNT concentrations above 1.0 wt.% led to agglomeration, reducing the mechanical properties of the composites. In the work of Liu et al. [131], CVD was utilized to coat spherical Ti-6Al-4V particles with CNTs, improving powder flowability and enabling the production of high-density specimens via selective laser melting with a tensile strength of 1162 MPa and elongation of 3.2%.

On the other hand, the PVD method has demonstrated effectiveness in depositing metallic coatings onto carbon nanostructures, enhancing their interaction with metallic matrices. In the work of Huang et al. [132], copper coatings were applied via PVD onto graphite films prior to vacuum hot pressing with an aluminum matrix. This approach achieved thermal conductivity up to 805 W/(m·K) in plane and 50 W/(m·K) out of plane due to improved wettability and reduced porosity at the Cu/Al interface. Additionally, the formation of Al_2_Cu phases contributed to efficient heat transfer and enhanced composite strength.

In another study, Bhat et al. [133] used the EPD method to deposit CNTs onto 50 μm-thick copper foils, followed by hot pressing and rolling. The composites demonstrated an increase in yield strength to 183 MPa and elongation to 30.9%, attributed to dislocation blocking and uniform CNT distribution between copper layers. Similarly, Zhang et al. [134] applied EPD to deposit CNTs onto magnesium plates before accumulative roll bonding (ARB). Composites with an 8 min CNT deposition time achieved electromagnetic shielding effectiveness up to 95 dB in the 8.2–12.4 GHz range. This improvement was attributed to multiple reflections of electromagnetic waves in the layered structure and uniform CNT distribution.

Laser deposition (Laser engineered net shaping, LENS^TM^) also allows for the application of coatings with minimal thermal deformation of the matrix and high structural control. In the study by Bhat et al. [135], laser deposition was used to create Cu-10Sn composites reinforced with 12 vol% CNTs. The mechanical properties of the composites significantly improved: the Young’s modulus increased by 82%, and yield strength by 26%. Microstructural analysis revealed uniform CNT distribution and minimized reactions between the matrix and CNTs due to high cooling rates.

Deposition methods are effective approaches for creating metal matrix composites reinforced with carbon nanotubes. However, these methods face limitations such as the need for vacuum conditions and weak substrate adhesion, which can lead to insufficient interfacial bonding. Hybrid methods, such as integrated laser and sol-gel deposition proposed in [136], can address these issues. This approach reduces vacuum requirements, improves coating adhesion through metallurgical bonding, and minimizes microstructural defects. Optimizing parameters such as laser power, deposition temperature, and sol-gel composition facilitates the formation of dense and wear-resistant coatings with high hardness and low friction coefficients.

### 2.4. A Brief Review of the Methods for Producing Al-CNT Composites

Methods for producing aluminum matrix carbon nanotube (Al-CNT) composites are diverse and produce materials with a wide range of properties. However, each method has unique challenges and limitations that require careful optimization to improve the efficiency and quality of the resulting composites. Table 1 shows the Al-CNT composite fabrication methods, their effect on the composite structure, and the advantages and limitations of the methods.

There are several suggestions for future research in the field of aluminum matrix composites. Perhaps more emphasis should be placed on new combined reinforcement materials such as carbon nanotubes, graphene and nanocellulose fibers to improve composite properties. Research into advanced manufacturing techniques, including hybrid manufacturing based on additive processes and spark plasma sintering, can improve the quality and efficiency of AMC production. The optimization of processing parameters such as temperature, pressure and time is essential to obtain composites with the desired properties. To achieve this, modeling techniques can be used to predict the behavior of composites under different loading conditions and optimize their design. Further research into the production of Al-CNT composites should focus on the integration of different processing methods and the application of hybrid approaches, which will provide composites with improved performance characteristics that meet the requirements of modern technologies and industrial applications.

## 3. Properties of Al-CNT Composites

### 3.1. Mechanical Properties

The wettability issue between CNTs and aluminum, as well as their incompatibility in terms of surface tension, limits interfacial adhesion and complicates the creation of Al-CNT composites [137]. To address this, interfacial reactions such as the formation of Al_4_C_3_ are being actively studied. While these reactions can improve adhesion, they reduce strength and ductility due to the brittleness of Al_4_C_3_ and the degradation of CNT structures [138]. Alternative approaches, such as CNT surface modification and the use of interfacial layers, present promising solutions for improving adhesion and mechanical properties. For example, using SiC as a coating has shown positive results in enhancing the wettability and distribution of CNTs in the aluminum matrix [139,140]. Increasing the volume fraction of SiC leads to higher hardness values, with the best result observed at a 12% volume fraction of SiC. This can be attributed to the strengthening effect of hard SiC particles and their inherently higher hardness. The higher the volume fraction of SiC, the better the distribution of particles within the aluminum matrix [140].

**Figure 8:**
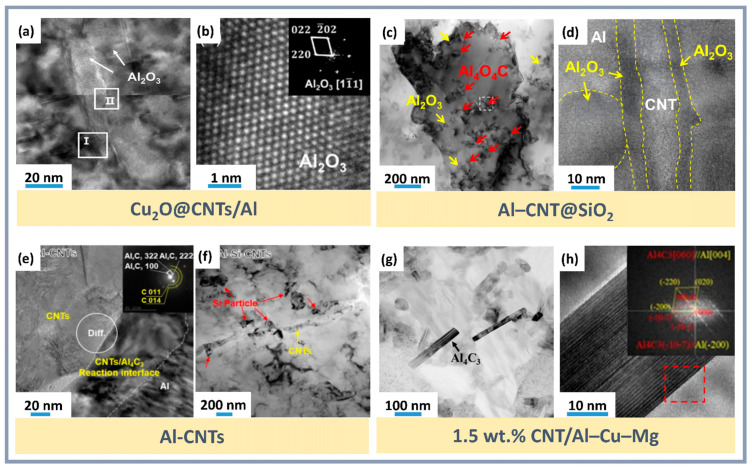
TEM images of (**a**) the intragranular Al_2_O_3_ whiskers and (**b**) area I recorded in (**a**), showing the Al_2_O_3_ and Al_2_O_3_-Al interface [138], (**c**) Al-Al_4_O_4_C fabricated at 610 °C and (**d**) a continuous Al_2_O_3_ layer on the surface of the CNT fabricated at 570 °C [141], (**e**) interface and (**f**) microstructure [71], (**g**) morphology and (**h**) high-resolution image of Al_4_C_3_ phase [118].

Additionally, employing metallic nanoparticles to create interfacial layers such as CuAl_2_ or Al_3_Ni improves mechanical characteristics, although excessive layer thickness can limit bonding efficiency. Thermite reactions, such as Al-CuO or Al-Fe_2_O_3_, form Al_2_O_3_ nanoparticles and intermetallics, enhancing interfacial bonding and increasing composite strength. For instance, a thermal reaction method for producing Cu_2_O@CNTs improved the mechanical properties of composites through the formation of an Al_2_O_3_ interfacial layer (Figure 8a,b) [138].

However, CNT agglomeration and reduced ductility remain issues in Al–CNT composites. Studies indicate that CNT agglomerates lower the reinforcing effect and plasticity of composites [142]. To mitigate this, three strategies are proposed: penetration of reinforcement into Al grains [143], use of dense nanoparticles to improve stress distribution [144], and creation of heterostructured composites to enhance plasticity [145]. Despite the success of these strategies, managing the process and ensuring uniform particle distribution remain challenging.

Increasing CNT content can improve stiffness, but poor dispersion may lead to a reduction in the modulus of elasticity due to agglomeration. For example, ultrasonic dispersion improved mechanical properties at 1.00 vol% CNTs, while higher CNT content led to reduced strength due to the formation of large CNT clusters [112]. Achieving optimal mechanical properties requires precise control over dispersion and production conditions, as demonstrated in studies using ultrasonic dispersion, which showed improvements in tensile strength and stiffness.

The authors of [140] also note that CNT volume fractions between 0.25 and 1 wt.% have a minimal impact on compressive strength. However, increasing the CNT volume fractions results in noticeable softening, which could be related to the void formation effect of the CNTs. Furthermore, introducing CNTs into the microstructure of Al composites led to gradual and continuous grain refinement until the CNT content reached 0.75 wt.%. After this point, at a 1 wt.% CNT content, significant agglomeration occurs, which reduces their effect on the structure and eventually results in the formation of large grains similar to those in pure aluminum.

Moreover, stress distribution and Young’s transverse modulus in aluminum composites with CNTs vary with changes in the distance between CNT bundles. Increasing the distance from the CNT bundle center to the control point reduces stress [146]. Therefore, synthesis methods for Al-CNT composites must effectively increase this distance.

Heterostructured composites produced via extrusion have also shown promising results. These materials feature regions with coarse-grained and fine-grained structures, enhancing mechanical properties through dynamic recrystallization of aluminum and heterodeformation-induced strengthening. For example, a CNTs/Al composite with a fine-grained zone and a quasi-continuous coarse-grained zone exhibited high tensile strength (549 MPa) and ductility (11.2%), surpassing values reported in the literature [101].

**Figure 9:**
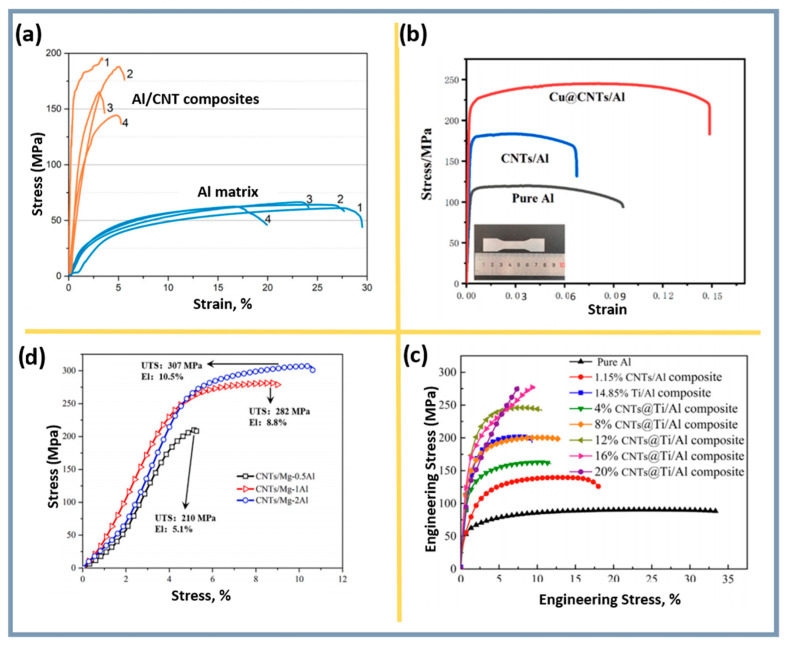
Tensile stress–strain curves of various composites: (**a**) [147], (**b**) [144], (**c**) [79], (**d**) [104].

A study [147] revealed that nanocomposites produced via powder metallurgy with 1.00 vol% reinforcing components and ultrasonic dispersion showed a 185% increase in yield strength due to load transfer mechanisms (Figure 9). However, the presence of CNTs at grain boundaries reduced ductility, as confirmed by EBSD observations and tensile testing results. The nanocomposites demonstrated improved hardness and Young’s modulus, although CNT clustering reduced plasticity. Microstructure and Vickers hardness analyses explained this behavior: Al samples showed high hardness due to high dislocation density, while Al-CNT nanocomposites exhibited increased dislocation density in undamaged areas.

Kumar et al. [26] demonstrated that increasing the temperature from 550 °C to 600 °C improved microhardness but that higher CNT concentrations reduced density while enhancing microhardness. Statistical analysis confirmed that CNT concentration had the greatest impact on microhardness and density.

Other studies, such as those by Xiang et al. [113] and Y. Li et al. [118], also reported improved mechanical properties of Al matrices reinforced with CNTs, with strengthening efficiency depending on failure mechanisms and temperature. Research shows that CNTs in composites enhance strength through increased dislocation accumulation and influence anisotropy, which is crucial under high-speed and high-temperature deformation conditions.

In Zuo et al. [44], the importance of temperature control for optimizing composite strength and ductility was emphasized. The results showed that increasing the temperature to 500 °C improved particle bonding and prevented grain coarsening, enhancing mechanical properties.

Minimizing CNT aggregation and ensuring proper nanoparticle distribution were discussed by Sasani et al. [58], where increased strength without plasticity loss was attributed to improved CNT dispersion in the matrix. Figure 9 illustrates the influence of CNT concentration and different composites on tensile stress–strain behavior.

In addition, Ziaei et al. [141] highlighted the significance of controlling interfacial reactions to achieve high mechanical properties, which can be effectively utilized in production processes.

Wan et al. [72] investigated the structural formation mechanisms in Al composites, such as CNTs-Al_2_O_3_, Al_4_C_3_, and CNTs. For flake powder, CNTs formed an oxide film that transformed into an interfacial layer, enhancing the composite. For granular powder, CNTs were embedded within granules, forming an Al-CNT composite. High-energy consumption resulted in Al_4_C_3_ formation, reducing reinforcement efficiency. Component effectiveness varied: RCNTs > RAl_4_C_3_ > RCNTs-Al_2_O_3_, with uncoated CNTs providing the highest strength.

**Figure 10:**
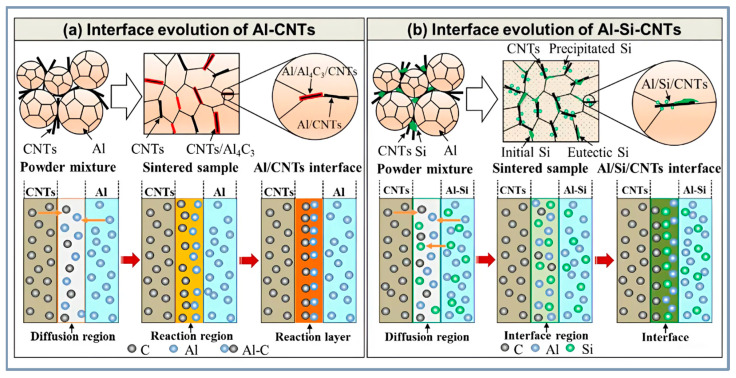
Evolution of the Al-Si/CNT composite interface: (**a**) Al-CNTs, (**b**) Al-Si-CNTs [71].

In [71], the addition of Si to Al-5Si-0.5CNTs increased the strength to 391 MPa with a ductility of 7.5%, improving load transfer from Al to CNTs. Strength increased by 79%, 69%, and 48% compared to pure Al, Al-0.5CNTs, and Al-5Si, respectively, emphasizing the importance of Si in enhancing strength and elasticity. Interface evaluation of composite is shown by the scheme in Figure 10.

Mg-compensated SiCp(CNT)/7075Al composites showed an improved balance of strength and ductility. Adding 1% Mg increased the ultimate strength to 651 MPa, while CNTs improved the ductility [112].

**Figure 11:**
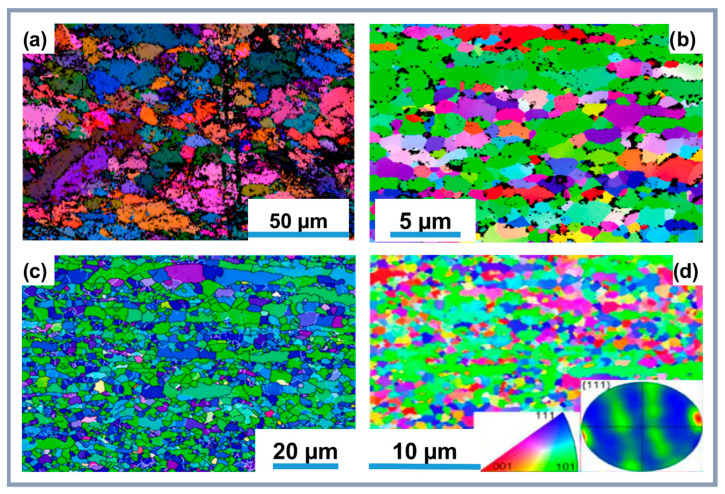
Grain information of (**a**) A383 Al-Si-Cu alloy matrix composites reinforced with 1.0 wt.% of chopped MWCNTs [119], (**b**) CNTs/Al-AR25 [143], (**c**) CNTs/2Al-Mg composite [79], (**d**) 0.5CNTs/5Si-Al composite, its phase and grain boundary distribution [143].

An A383-CNT composite produced via ORDC with 1.0 wt.% CNTs exhibited a 21% increase in tensile strength compared to monolithic A383 [119], confirming the method’s effectiveness (Figure 11a).

Therefore, the method of composite synthesis and the addition of components significantly affect the strength and ductility properties of the composites. A key factor is fine-grain strengthening (Hall–Petch effect), which is attributed to the reduction in grain size through the introduction of CNTs and other phases [118]. It is important to note that extrusion lines are visible in Figure 11, with grain sizes reduced due to the inclusion of CNTs [143]. The extrusion and heat treatment processes promote the formation of a fine-grained structure, improving interfacial adhesion and stress distribution. Dynamic recrystallization, which occurs post-extrusion, is especially effective in achieving a uniform microstructure [79,143].

In the Al-C system, an exothermic reaction occurs, forming the brittle Al_4_C_3_ phase, which reduces ductility. The addition of CNTs with copper and silicon improves adhesion at the interfacial boundaries and suppresses the formation of Al_4_C_3_. While Al_4_C_3_ increases strength by preventing fracture during tension and improving the bond between CNTs and the aluminum matrix, an excess of this phase leads to a loss of material ductility. For example, copper nanoparticles eliminate defects on the surface of CNTs, passivating them and preventing the formation of Al_4_C_3_ [138]. The addition of silicon to the Al-Si-CNTs system also prevents the formation of Al_4_C_3_ (Figure 8e), preserving the ductility of the composite [71].

**Figure 12:**
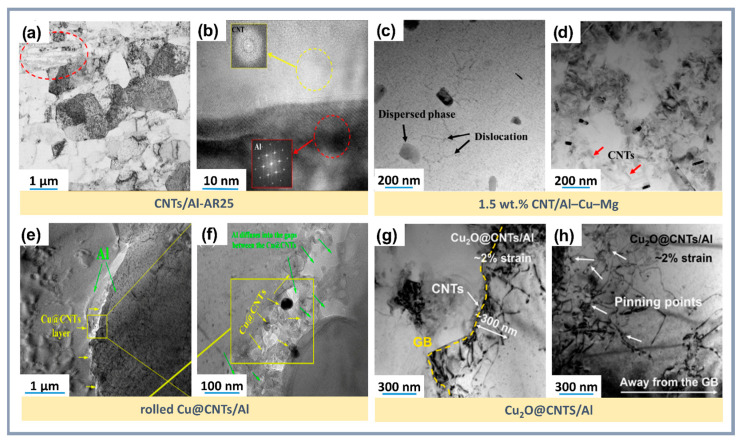
TEM images of composites: (**a**) CNT aggregations (bright field and circled by a red line) and (**b**) displays of the CNTs-Al interface [143], (**c**) dislocation and second phase ((Al,Cu)_3_Ti phase) distribution, (**d**) distribution and morphology of CNTs [118], (**e**,**f**) microstructure and Cu@CNTs dispersed at the interlayer place [144], (**g**,**h**) with strain deformation of ~2% [138].

At the Al_2_O_3_-CNTs interface, oxygen partially interacts with aluminum, forming a nanoscale Al_2_O_3_ layer on the surface of the CNTs, which reduces the likelihood of microcracks. Needle-like Al_2_O_3_ crystals (less than 20 nm in diameter, 40–120 nm in length) and CuAl_2_ particles within the grains capture dislocation loops and create a stressed state, contributing to strengthening [138]. Strong interfaces, such as Al_2_O_3_-CNTs and Cu@CNTs-Al (Figure 12e), provide reliable bonding between the matrix and strengthening components [144]. At high temperatures, chemically stable covalent bonds, such as Al_4_O_4_C, are formed, which significantly outperform Al_4_C_3_ in terms of hardness, stability, and resistance to hydrolysis. Phases Al_4_O_4_C (Figure 8c,d) [141] and Mg_17_Al_12_ (Figure 11c) [79] strengthen interfacial bonds, increase the elastic modulus, and prevent dislocation movement, thus improving mechanical properties.

Nanoparticles (Al_2_O_3_, Cu, Si) help trap dislocations, increase their density, and create internal stresses in the grains, significantly enhancing the composite’s strength. The thermal mismatch between the CNTs and the matrix further hinders dislocation movement. Retaining plasticity is achieved by activating non-basal dislocation systems and optimizing the distribution of CNTs. Processing methods (modification of CNTs, thermite reaction, ball milling, heat treatment) ensure a uniform dispersion of reinforcing materials. The use of the SPS method allows for the achievement of high composite density without the formation of carbides and intermetallics due to precise control of temperature, dwell time, and pressure [137,143]. A similar effect is achieved using multi-particles (ORDC), helping reduce porosity, form Al_2_O_3_, and improve mechanical properties [119]. Table 2 shows how the synthesis method and parameters affected the mechanical properties of the composite.

The addition of CNTs, nanoparticles, and other phases refines the aluminum matrix grains, providing additional strengthening. The uniform distribution of CNTs promotes efficient load transfer to the matrix (Figure 12). The removal of amorphous carbon and the creation of oxygen-containing functional groups on the surface of CNTs after acid treatment increase roughness and promote the nucleation of copper nanoparticles [138]. Silicon prevents the Al-C reaction and suppresses the formation of brittle phases [71]. The optimal CNT concentration (around 1.0 wt.%) ensures maximum mechanical properties, while exceeding this value leads to aggregation (Figure 12a), increased porosity, and deterioration of the properties [119]. Copper enhances the bonding between CNTs and the matrix, eliminates defects on the surface of CNTs, and improves the material’s strength [144]. SiO_2_ forms a stronger oxide layer on CNTs, improving their bond with the matrix [141]. Therefore, it is necessary to take into account the changes in the structure and, consequently, the properties of CNTs both during the synthesis of the composite and after it. However, in many cases, this issue remains insufficiently studied, despite noticeable changes in the morphology of CNTs, which serve as a reinforcing component of the composite and play a key role in its properties.

CNTs indeed cause significant distortions or internal deformations in composites, which result in the formation of numerous unidentified regions in EBSD images (Figure 11). However, the combined effect of CNTs, nanoparticles, and their interactions with the matrix generates a dispersion strengthening effect that enhances the elastic modulus, strength, and ductility. Interfacial reactions, such as the thermite reaction, lead to the creation of strengthening phases (needle-like Al_2_O_3_ crystals and CuAl_2_ particles), reinforcing the synergy between strength and ductility properties [138,143].

Al-CNT nanocomposites show significant improvements in mechanical properties, including strength and ductility. However, to optimize these properties, it is crucial to take into account factors such as temperature, CNT concentration, microstructural characteristics, and the interactions between the matrix and reinforcing particles. Future research should focus on addressing the decrease in ductility at higher strength levels by achieving more precise control over the material’s micro- and macrostructure.

### 3.2. Wear Resistance

Enhancing the wear resistance of aluminum-based composites reinforced with CNTs requires careful consideration of key factors such as CNT content, the ratio of reinforcing particles to the matrix, and operating conditions, including sliding speed, applied load, and distance traveled. A study [151] investigated the influence of these parameters using a fractional factorial Taguchi matrix, revealing that with an optimal composition (3% CNTs and a 50% component ratio), the wear rate could be reduced by 80%. However, the combined effects of load and composition on wear resistance remain complex and warrant further investigation.

It is well known that double-shot peening (DSP) significantly improves the wear resistance of Al/CNT-Cu-Mg composites [152]. Key improvements include increased hardness, the introduction of residual compressive stresses, reduced surface roughness, and refined grain size. These factors collectively decrease wear and enhance material durability. Notably, DSP-treated samples exhibit greater wear resistance than those subjected to conventional shot peening.

**Figure 13:**
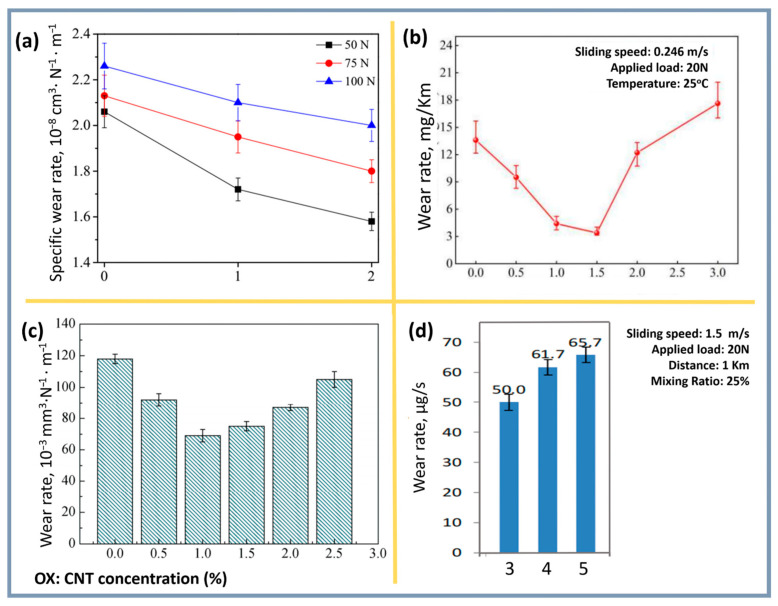
Variations in wear rate of composites: (**a**) Al6061-SiCp/CNT [103], (**b**) Al-Cu-Mg-Si/CNT [153], (**c**) Al-Si-10Mg/CNT [154], (**d**) Al-(Al-CNT) [151] with different CNT contents.

An important parameter is the wear rate (k), calculated based on the volume of material loss, which allows for a detailed evaluation of wear resistance across various composites:

k = ΔV/(P·L),(1)

here, P is the applied load, L is the sliding distance, and ΔV represents volume loss, typically expressed as mass loss (Δm) divided by the measured composite density [118]. This parameter underpins wear resistance assessments for materials such as Al-CNT composites and provides an evaluation of the influence of different factors (load in Figure 13a, CNT concentration in Figure 13b–d) on their wear resistance. For instance, with 1.5% CNT content in Al-Cu-Mg-Si composites, both the coefficient of friction (COF) and wear rate are minimized, making the material more resistant to wear under varying temperatures [153].

Optimal CNT content for improved wear resistance is also confirmed by other studies, showing that minimal wear rates are achieved at a 1.0–1.5% CNT content due to their uniform distribution and lubricating properties [110]. Increasing CNT concentration to 2% causes nanotube agglomeration, negatively affecting wear resistance.

In another study [154], the lowest wear rate was observed at a 1.0% CNT content, attributed to uniform distribution and the ability of CNTs to reduce matrix oxidation. However, at CNT contents above 1.0%, wear increased due to porosity and cracking. For optimal mechanical properties and wear resistance, the CNT content should range between 1.0% and 1.5%.

Adding up to 0.3% CNT reduces wear due to their lubricating properties and increased hardness, but excessive addition leads to agglomeration, increasing wear. For example, at 0.3% CNT, wear is minimal, while it is significantly higher for pure AlSi10Mg. At fixed loads or sliding speeds, wear increases with the rise in these parameters [110].

CNTs also form a lubricating film that reduces friction and protects the surface from further wear. This film reduces mechanical material removal and wear caused by micro-plowing. Sarkar et al. [155] demonstrated that adding 0.5% CNTs to AA6061 composites improved wear resistance and reduced the coefficient of friction, decreasing specific wear rates by 77% compared to untreated aluminum (Table 3).

Simultaneously, Sharma et al. [156] found that the optimal content of multi-walled CNTs (MWCNTs) in Al composites is 0.5%, which reduces mass loss due to wear by 75%. However, increasing MWCNT content to 1.0% decreases wear resistance due to agglomeration, which weakens adhesion and increases wear.

The main changes affecting the wear resistance of the composite are related to the formation of protective phases and the improvement of the barrier properties of the coating. The authors of [157] report that CNTs can act as separators, preventing direct contact between the composite and the opposing surface. This leads to reduced friction and wear. CNTs prevent excessive surface damage by reducing the wear intensity. When CNT particles are removed from the surface, they can also function as self-lubricating components, further reducing the friction coefficient and wear. Additionally, a rolling effect of CNTs on the surface is observed, where the removed CNT cylinders easily roll between the contacting surfaces, reducing friction.

Furthermore, the SPS method also positively affects the wear resistance of the composite [146,157]. The increased density of the material due to reduced porosity at high temperatures lowers the likelihood of microcrack and pore formation, which can serve as initiation points for wear and failure. This structural improvement also contributes to the overall enhanced wear resistance of the material.

Thus, achieving maximum wear resistance in aluminum-CNT composites requires careful selection of the CNT content, processing parameters, and microstructural characteristics, such as grain size and particle distribution. These factors ensure an optimal balance of strength and plasticity during operation. Additionally, interaction mechanisms between CNTs and the matrix, as well as operational conditions like sliding speed and applied load, must also be considered (Figure 13).

**Table 3:** Al-CNT composites and their wear resistance properties.

Composite	PM^1^	SP^2^	WV^3^	WR^4^	COF^5^	Comms	R^6^
Al_2_O_3_-ZrO_2_/CNT	Atmospheric plasma spraying	40 KW, 57.20 V	-	0.9·10^−6^	0.57	Synergistic effect of ZrO_2_, CNT and bimodality helps in enhancing wear resistance.	[150]
AA6061-0.5 wt% CNT	Squeeze-casting	750 °C, 2 min, 100 MPa	n/a	9.2·10^−4^	0.19	When the load was increased to 10 N, COF increased to 0.41, but at 15 N, it decreased to 0.34, and SWR decreased to 7.9·10^−4^ mm^3^·N^−1^·m^−1^ due to the self-lubricating effect of CNT.	[155]
Al_71_Ni_14.5_Co_14.5_/CNT poly-quasicrystal	MA and SPS	950 °C, 10 min, 80 MPa	n/a	1.0·10^−4^	n/a	21.5% reduction in compressive strength, although the compressive strength remained above 1.1 GPa at 600 °C.	[157]

PM^1^—Production method; SP^2^—Synthesis parameter; WV^3^—Wear volume, mm^3^; WR^4^—Specific wear rate (k), mm^3^·N^−1^·m^−1^; COF^5^—Coefficient of friction; R^6^—Reference.

### 3.3. Corrosion and Erosion Resistance

The increased resistance to corrosion and erosion makes CNT-reinforced composites suitable for operation in aggressive environments [158]. To fabricate metal-based composites with enhanced mechanical and corrosion-resistant properties, various methods, such as PM [159,160], laser additive manufacturing [161], FSP [162], coating applications [163], and SPS [164], are employed. These techniques enable the production of composites with superior properties compared to pure metals.

One key indicator of corrosion resistance is the corrosion rate (CR), calculated using the following formula:

(2) $$\mathrm{CR}=K\;\cdot \;\frac{i_{\mathrm{corr}}}{p}\;\cdot \;\mathrm{EW},$$

where CR is measured in mm/year, i_corr_ is the current density (µA·cm^−2^), K = 3.27·10^−3^ mm·g·µA^−1^, p is the density (g·cm^−3^), and EW is the equivalent weight of the material. This formula was used in [165] to evaluate the corrosion resistance of Al-CNT composites (Table 4).

CNTs contribute to improved corrosion and wear resistance of aluminum, making these composites more suitable for extreme conditions [166]. In [95], the effect of adding MWCNTs to aluminum composites on their microstructure and corrosion properties was investigated. A mixture of aluminum powder and CNTs was processed in a planetary ball mill, which improved the uniform distribution of CNTs and the composite’s corrosion resistance. However, increasing the CNT content to 5% deteriorated the distribution and reduced corrosion resistance. The results showed an increase in polarization resistance from 1.28 to 3.60 Ω·cm^2^ with milling time extended to 4 h, but polarization resistance decreased to 2.76 Ω·cm^2^ at a 5% CNT content. Additionally, the study found that the corrosion resistance and mechanical properties of Al–CNT coatings were significantly higher than those of coatings without CNTs due to fewer and smaller pores.

After EDT treatment in a dielectric fluid with MWCNTs [165], nanostructures and intermetallic compounds, carbides, and silicides are formed on the material’s surface, significantly enhancing its mechanical strength and resistance to wear and corrosion. These changes help strengthen the material’s surface layers, reducing its susceptibility to corrosion. However, the introduction of MWCNTs into aluminum matrices improves mechanical properties but deteriorates corrosion characteristics, as CNT agglomerates may promote more active localized pitting corrosion in areas of their concentration.

While introducing CNTs into a metallic matrix can significantly enhance mechanical properties, differences in the potential between the reinforcing particles and the metallic matrix, along with the heterogeneous microstructure of different composite regions, complicate the corrosion mechanism. This mechanism includes galvanic, pitting, and uniform corrosion [95,167,168]. The morphology of CNTs plays a crucial role in their distribution within the matrix, influencing both the mechanical and corrosion behavior of composites [169].

It is worth noting that adding CNTs to aluminum–silicon composites does not create a passive zone, which means active corrosion occurs. However, CNTs improve corrosion characteristics by increasing the corrosion potential and reducing I_corr_, indicating better corrosion resistance. The composite with 0.8% CNT has the highest polarization resistance and improved corrosion resistance. At 0.2% CNT, galvanic corrosion is observed, causing the destruction of the oxide film and allowing chloride ions to reach the Si particles, which reduces resistance. At 0.8% CNT, pitting corrosion occurs with smaller pits and a lower corrosion rate, owing to the better distribution of CNTs, which slow down the corrosion process by preventing the penetration of aggressive ions [149].

**Figure 14:**
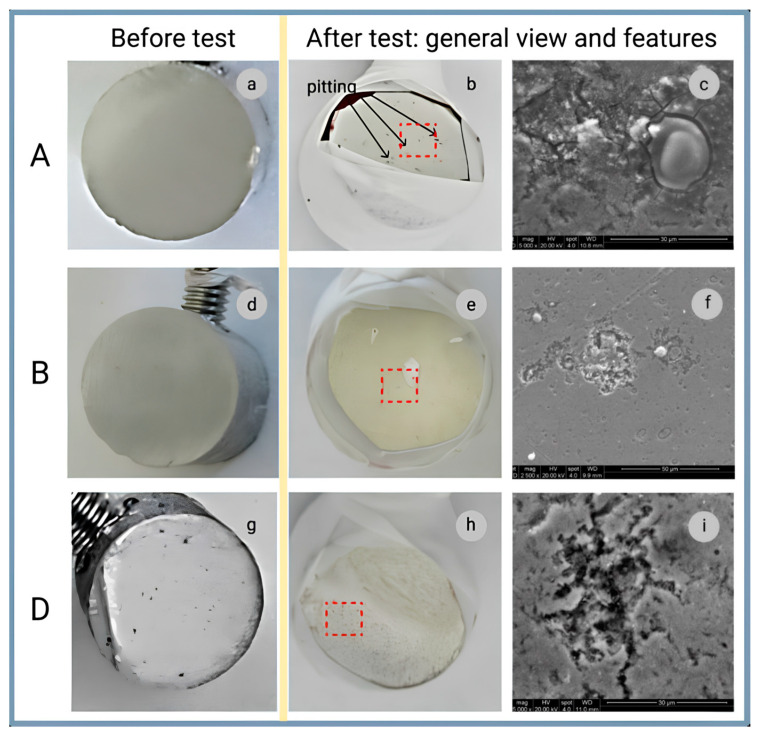
Corrosion behavior of aluminum samples: (**a**–**c**) pure aluminum 1070 before and after testing; (**d**–**f**) Al + 0.25 wt.% MWCNTs before and after testing; (**g**–**i**) Al + 0.5 wt.% MWCNTs before and after testing [120].

The study by Popov et al. [120] illustrates in detail the corrosion processes in Al–MWCNT composites with varying CNT content (0.25 wt.% and 0.5 wt.%, shown in Figure 14b,d, respectively) compared to pure aluminum (Figure 14a), which served as the reference for corrosion resistance evaluation. Figure 14a,d,g shows the initial surfaces of the samples before testing, while Figure 14b,e,h depicts the surfaces after testing, where slight pitting corrosion is observed. Figure 14c highlights cracking in the corrosion zones. Magnified images (Figure 14f,i) reveal more intense pitting corrosion in regions with CNT clusters. This is explained by the galvanic effects at the matrix-CNT interface, which make composites more susceptible to pitting corrosion compared to pure aluminum. Pitting corrosion remains a key degradation mechanism for aluminum alloys and their composites [168].

Increasing the surface area of powders through mechanical milling improves the adhesion of CNTs and promotes better distribution of nanotubes in the matrix, which, in turn, can enhance corrosion resistance. These nanotubes can act as a barrier, reducing the contact between the material and the external environment, preventing the penetration of corrosive agents into the metal. At the same time, CNTs can alter local electrical properties, which may also increase corrosion resistance [149,157].

Oxidation of the powder surface during prolonged milling forms a protective Al_2_O_3_ film, which can reduce corrosion activity if oxidation is controlled. This also helps improve the material’s protection against the penetration of aggressive ions and substances into the metal’s structure. This leads to a reduction in material degradation [149,165].

The formation of phases such as aluminum phosphide and aluminum phosphate can also positively influence the corrosion resistance of the composite [157]. These phases can serve as protective coatings, preventing the penetration of corrosive agents (e.g., water or oxygen) into the material’s structure. Aluminum phosphides and phosphates have known anticorrosive properties that can enhance protection against corrosion, especially in aggressive environments.

Additionally, dislocations formed between CNTs and aluminum powders can affect the microstructure, mechanical properties, and corrosion characteristics of the materials. Thermal treatment can modify the microstructure and improve composite properties [170].

Thus, the microstructure and distribution of CNTs in aluminum matrices are critical for enhancing the mechanical and corrosion resistance of composites. These factors are essential for developing new materials with improved corrosion resistance, suitable for operation in high-humidity and aggressive environments.

**Table 4:** Al-CNT composites and their corrosion resistance properties.

Composite	PM^1^	SP^2^	CP^3^	CD^4^	CR^5^	Comms	R^6^
Al-Si10-Mg/0.8%CNT	SPS	540 °C, 18 min, 40 MPa	−324	−6.34	n/a	Composite has the large radius of circle and large polarization resistance,	[110]
Al1070-0.5%MWCNTs	HP die casting	760 °C, 200 MPa	−787 (0.5 h)	0.02	n/a	Yield strength is 104 MPa, tensile strength is 132.2 MPa	[120]
6Al-Ti−4V/CNT	Taguchi	Dielectric liquid treatment	3.51	1.83	0.03	Improved surface hardness to 10 times (4452.5 HV),	[131]
Al_71_Ni_14.5_Co_14.5_/CNT poly-quasicrystal	MA and SPS	950 °C, 10 min, 80 MPa	−324	0.20	n/a	The QC sample exhibited the lowest I_corr_ at 0.12 µA·cm^−2^	[157]
Al-2GAg,NPs/4%CNTs	SPS	580 °C, 50 MPa, 5 V, 300 amps	−170	43.27	0.25	Corrosion resistance is 163.7 Ω.cm^2^	[171]

PM^1^—Production method; SP^2^—Synthesis parameter; CP^3^—Corrosion potential (E_corr_), mV; CD^4^—Current density (I_corr_), µA·cm^−2^; CR^5^—Corrosion rate, mm/year; R^6^—Reference.

### 3.4. Electrical and Thermal Conductivity

In the energy sector, significant energy losses occur during the transmission and distribution of electricity due to technical issues such as corrosion, mechanical damage, and conductor oxidation. These losses lead to substantial financial costs and a decrease in the overall efficiency of energy systems. Developing new materials for conductors with enhanced properties, such as high electrical conductivity, strength, and corrosion resistance, is a critical task for improving energy transmission efficiency. This is especially relevant for countries with high technical losses in power grids [172,173,174].

A study [175] employed electrophoretic deposition of copper with functionalized carbon nanotubes (Cu/f-CNT) on aluminum wires to improve their electrical properties. This method enhances CNT dispersion and improves conductor conductivity by 18% compared to conventional aluminum wires. Doping CNTs with iodine increases conductivity by enhancing surface charge and creating conductive channels. The study also demonstrated that the conductor remains stable when heated up to 50 °C. This approach offers the potential to develop lightweight, high-performance conductors suitable for high-temperature and aggressive environments, such as power transmission lines and electrical connections.

One of the main challenges in improving the electrical conductivity of Al-CNT composites is the annealing process during synthesis. At elevated temperatures, Al_4_C_3_ forms at the interface, which improves mechanical strength through strong bonding between aluminum and CNTs [141]. However, excessive Al_4_C_3_ reduces strength due to its brittleness and negatively affects the conductivity of the aluminum matrix. Controlling the reaction that leads to Al_4_C_3_ formation is difficult [176]. Nevertheless, researchers [68] have developed a composite combining good hardness with high conductivity by regulating the size and dispersion of nanoscale Al_4_C_3_ grains (Table 5). Their study demonstrated that using a pure nanoaluminum powder enables highly dispersed CNTs with controlled interfacial boundaries between aluminum and CNTs, achieved by reducing the oxide layer on the aluminum surface. Fine Al_4_C_3_ grains at the Al-CNT interface during low-temperature annealing (663 K) increase composite hardness while preserving electrical conductivity by minimizing oxide layers at the Al/Al interface. As a result, Al-CNT composites exhibit conductivity 33 times higher than Al–nanocarbon composites with similar hardness. Furthermore, adding CNTs improves the conductivity of aluminum conductors, especially in combination with copper and iodine doping, by increasing the number of conductive channels and thermal stability.

In another study [177], adding 3% CNTs enhanced the conductivity and structural stabilization of CNTs-LiFePO_4_-Al foam cathodes, achieving excellent results in cyclic tests with a capacity retention of 82% after 2000 cycles, 76% after 5000 cycles, and 69% after 9000 cycles.

Thus, the addition of CNTs to Al–CNT composites can significantly improve conductivity, but maintaining a balance between mechanical strength and conductivity is crucial. However, the limited number of studies on the effects of CNTs on the electrical properties of composites highlights the need for further exploration to unlock the full potential of Al–CNT composites in this area.

For improved thermal conductivity (TC) and reduced thermal expansion (CTE) in device miniaturization, Al matrices with CNTs address thermal mismatch with semiconductors while maintaining high strength. Table 6 presents the thermal properties of Al–CNT composites.

According to a study [178], adding 2 wt.% CNTs to Al-20Si powder provides an optimal combination of strength, low CTE, and TC. Uniform CNT distribution, crucial for improved properties, is hindered by strong Van der Waals forces and the high aspect ratio of CNTs. Excessive CNT content leads to agglomeration, worsening thermal properties. This is corroborated by reduced oxygen content in Flake Al-20Si-3CNT, indicating CNT cluster formation and reduced contact area with aluminum. During mechanical alloying, Al-20Si particles flatten, CNTs disperse, and silicon particles break down, forming a microstructure of ultrafine silicon surrounded by CNTs. Oxygen adsorption on fresh surfaces forms amorphous Al–C–O or crystalline Al_2_O_3_ structures, stabilizing silicon particles and preventing their growth.

Consequently, Flake Al-20Si-3CNT composites achieve a yield strength of ~235 MPa, 2.5 times higher than extruded Al-20Si (~90 MPa). This strength improvement is attributed to a reduced Al grain size, silicon fragmentation, and increased grain boundaries, which inhibit dislocation movement. Reinforcement by CNTs, Al_2_O_3_, Al_4_C_3_, and silicon particles is due to strong interfacial bonding with the aluminum matrix, reducing plastic deformation. Nanoparticles further enhance strength through the Orowan strengthening mechanism.

TC and CTE depend on six factors: silicon, Al_2_O_3_, Al_4_C_3_, CNTs, phase boundaries, and dislocations. Reinforcement by Si, Al_2_O_3_, and Al_4_C_3_ and increased interfacial thermal resistance reduce TC despite the high thermal conductivity of CNTs. Al_4_C_3_ formation at CNT-Al interfaces partially restores the CNT effect. Low-CTE phases (Si, Al_2_O_3_, Al_4_C_3_, CNTs) constrain Al matrix thermal expansion, reducing the overall CTE (~16.2 × 10^−6^ K^−1^). Uniform CNT distribution and reduced Si size enhance this constraint.

A study [183] indicates that adding CNTs to Al increases the critical conductivity temperature by 50 K (up to 800 K). The activation energy for sintering CNT-Al is 5.53 kJ·mol^−1^, 55% higher than for pure Al (3.57 kJ·mol^−1^), indicating slower sintering rates.

Another study [145] notes a sharp decrease in thermal conductivity when CNT content reaches 2.0 wt.% (3 mm thickness) due to CNT cluster formation, reducing phonon mean free paths and thermal conductivity.

The 3D Representative Volume Element (RVE) model plays a crucial role in studying the thermal expansion of Al-CNT composites, accounting for anisotropy, CNT volume fraction, and component interfaces. This model is necessary for a precise analysis of CNT configuration effects on CTE in composites [182]. Predicted TC and other composite information is shown in Table 6. Traditional theoretical models often fail to consider the complex microstructure, orientation, and distribution of CNTs within the matrix. RVE modeling enables accurate predictions of composite properties, improving design and manufacturing processes where conventional testing methods may be inefficient.

## 4. Challenges and Further Prospects of Al–CNT Composites

Over the past decades, Al-CNT composites have been a focal point of extensive research due to their remarkable properties, including high strength-to-weight ratios and excellent conductivity. Most studies have concentrated on synthesis methods, processing techniques, microstructural characteristics, and mechanical properties. However, several challenges remain, hindering the broader adoption of these materials in industrial applications.

Key obstacles include the high cost of CNT production, agglomeration issues, and difficulties in achieving a uniform dispersion within the aluminum matrix. These factors significantly impact the scalability and cost effectiveness of Al-CNT composite fabrication. Furthermore, limitations in current fabrication technologies, such as achieving consistent interfacial bonding and mitigating the formation of brittle phases like Al_4_C_3_, continue to pose challenges. Addressing these issues requires advancements in processing methods and a deeper understanding of the interaction mechanisms between CNTs and the aluminum matrix.

Key challenges include the high cost of CNT production, agglomeration issues, and difficulties in achieving uniform dispersion within the aluminum matrix. To address these problems, methods such as HEBM, FSP, and SPS should be considered, as they allow for an even distribution of CNTs and help prevent a reduction in their reinforcing properties. However, to avoid damaging the CNT structure and prevent the cold welding of particles during synthesis, process agents like PCAs should be used. It should be noted that in most cases, studies have shown that the optimum CNT content for improving composite properties is between 0.5 and 1.5%, depending on the use of other additives, processing conditions and synthesis method. Additionally, to improve the quality of the bond between CNTs and the aluminum matrix, degassing in an inert environment can be employed to remove oxide films before mixing.

Furthermore, limitations in current fabrication technologies, such as achieving consistent interfacial bonding and preventing the formation of brittle phases like Al_4_C_3_, remain challenges. Addressing these requires advancements in processing methods and a deeper understanding of the interaction mechanisms between CNTs and the aluminum matrix. For example, decorating CNTs with aluminum oxide improves interfacial bonding and reduces Al_4_C_3_ formation, while lowering the synthesis temperature can help inhibit reactions between CNTs and aluminum.

These factors significantly impact the scalability and cost effectiveness of Al-CNT composite fabrication. The scalability issue can be addressed by combining powder metallurgy with additive manufacturing or chemical deposition, and molecular mixing for a uniform CNT distribution at the molecular level.

Additionally, studying the properties of CNTs during and after synthesis is necessary, particularly how their reinforcing properties change with structural alterations. To further enhance the mechanical and thermal properties of composites, it is important to investigate the exact interactions between CNTs, aluminum, and additives such as Si or Cu. Research into new additives that can stabilize phase transitions and improve mechanical and corrosion properties, as well as the introduction of new processing methods like laser deposition or sol-gel synthesis, to improve bonding between CNTs and the aluminum matrix, are also crucial.

Despite these challenges, Al-CNT composites have already proven to be valuable across various industries due to their exceptional properties. Their high strength-to-weight ratio is particularly advantageous in aerospace applications [184,185], where reducing weight without compromising structural integrity is essential. For instance, these composites can be utilized in structural components such as fuselages and the wing panel, contributing to improved fuel efficiency and reduced launch costs. They are also promising materials for spacecraft construction [186], where their use in satellites and space vehicles can decrease payload weight while enhancing radiation resistance.

Similarly, in the automotive industry [155,187], the lightweight and high-strength characteristics of Al-CNT composites improve vehicle performance by reducing fuel consumption and supporting environmental sustainability. For example, these materials are ideal for manufacturing lightweight yet durable body panels and chassis components, significantly reducing vehicle weight and boosting energy efficiency.

In the field of electronics and energy storage, Al-CNT composites offer high electrical conductivity and stability, which enhances the performance and reliability of devices such as batteries and capacitors [188]. Their application in this area enables faster response times and improved charging stability. Additionally, their lightweight and robust properties have opened up opportunities in construction, sports equipment production, and other industries requiring materials with similar performance characteristics.

Looking ahead, the unique properties of Al-CNT composites are likely to foster further innovation and expand their use in cutting-edge technologies, such as energy systems and advanced materials for electronics [189]. Continued research and development will undoubtedly strengthen their position in diverse industrial applications.

## 5. Conclusions

This study examines and analyzes various synthesis methods for aluminum–carbon nanotube (Al-CNT) composites, including powder metallurgy, diffusion and reaction bonding, as well as CNT surface modification. The impact of adding CNTs to the aluminum matrix on the composite’s properties is also discussed. The application of carbon nanotubes in aluminum composites is a promising direction due to their outstanding mechanical, thermal, and electrical properties.

A key factor that determines the operational characteristics of the composites is the uniform dispersion of CNTs and the strength of the interfacial bonding. Therefore, the synthesis method plays an important role in the formation of composite properties. Synthesis conditions affect the amorphousness of the CNTs, the distribution of additives, the formation of different phases, and interfacial layers due to thermal reactions, which can either improve or degrade specific material properties. Controlling the distribution of these phases is also essential to achieve an optimal balance between the composite’s strength and plasticity.

At the same time, there are certain challenges in synthesizing high-quality Al-CNT composites and implementing these methods in large-scale production. Addressing these challenges will contribute to the improvement in Al-CNT properties and the expansion of their applications. Therefore, future research should focus on enhancing the interactions between CNTs and the aluminum matrix, optimizing phase transitions, developing new additives, and improving the scalability of the process. This will open new opportunities for the use of Al-CNT composites in various industries and increase their economic efficiency.

## Figures and Tables

**Table 1:** Methods for fabricating Al-CNT composites and their characteristics.

Method	Influence on Morphology and Structure	Advantages	Limitations
PM	Uniform CNT distribution in the aluminum matrix; low temperatures prevent CNT agglomeration; oxide formation on powder surfaces may reduce Al-CNT bonding but can enhance properties in some cases.	Low-temperature processing; excellent microstructure control; low cost and flexibility; scalable for large-scale production.	Oxide layers may weaken composite strength; diffusion issues at high temperatures; inefficiency with high CNT content.
SPS	Enhanced strength via carbon “nails”; formation of barriers preventing CNT degradation but reducing interfacial bonding with aluminum; fine-grained microstructures with controlled porosity.	Reduced processing time; preservation of CNT properties; ability to create various microstructures; improved densification at lower temperatures.	Requires meticulous preparation; equipment demands (vacuum and inert atmospheres); formation of oxide films.
HEBM	Improved dispersion uniformity; reduced agglomeration; increased aluminum surface area for effective CNT interaction; potential for nanostructured composites.	Enhanced mechanical properties; customizable milling conditions; effective for breaking down CNT clusters.	High energy consumption; risk of CNT damage; requires precise parameter adjustment to avoid agglomeration.
HP/HE	Grain elongation increases anisotropy; improved toughness by crack redirection; formation of hybrid phases (SiCp/CNT) enhances fracture energy.	Increased yield strength and toughness; grain growth suppression; suitable for producing dense bulk components.	Mechanical anisotropy; formation of Al_4_C_3_ phases may weaken CNTs; high energy demands; precise control required.
EPD	Uniform CNT coating on metallic particles or substrates; formation of thin films with controlled thickness and dispersion.	Simple and scalable process; low-cost equipment; good control over coating thickness and uniformity.	Requires conductive substrates; adhesion issues without post-treatment; potential agglomeration.
CVD	Direct growth of CNTs on metallic powders; strong interfacial bonding through controlled reactions; uniform CNT distribution.	High purity and crystallinity of CNTs; controlled growth parameters; suitable for hybrid reinforcement strategies.	Requires high temperatures and vacuum; risk of CNT degradation; formation of unwanted carbide phases (Al_4_C_3_).
PVD	Thin metallic coatings on CNTs or substrates; improved interfacial bonding and wettability; controlled coating thickness.	High precision and purity; good adhesion; minimal thermal damage to CNTs; suitable for creating interfaces.	Requires vacuum; limited scalability for bulk production; high equipment cost; difficulty coating complex shapes.
Laser deposition	Dense, defect-free coatings; enhanced bonding between CNTs and matrix; minimal heat-affected zone.	Rapid processing; high precision; minimal thermal distortion; suitable for surface repairs and reinforcements.	High equipment cost; risk of porosity and cracks; requires precise control of laser parameters.

**Table 2:** Mechanical properties of Al-CNT composites fabricated by different methods.

Composite	PM^1^	SP^2^	YS^3^	TS^4^	E^5^	YM^6^	HV^7^	Comms	R^8^
Al-Al-Mg/CNT	ARB	375 °C,1 h	462.0	-	-	-	126.0 (10 kg)	Energy absorption capability is 19.2 MPa	[58]
Al-5Si-0.5CNTs	SPS	577 °C, 1 h, 30 MPa (a vacuum of 5 Pa)	320.0	391.0	7.5	91.5	-	Load transfer is 86 MPa	[71]
Al-10%SiCp/2%CNT	SPS	560 °C, 5 min, 50 MPa	178.5	249.5	6.5	-	159.5	The relative density is 99.1%	[103]
Al-CNT@ Ti	SPS	590 °C, 40 min, 50 MPa	-	284.0	10	-	~145	Increased efficiency of Al-CNT activities	[104]
Al-Cu-Mg/CNT	HP/HE	520 °C, 1 cm	456.7	480.4	11.9	-	-	Load transfer of 1.5% CNT is 7.0 MPa	[118]
Al-Si-Cu/CNT	ORDC	200–680 °C, 80 MPa, 32%, 20 mm	150.1	258.5	~1.8	-	157.9	The wettability has increased due to the improved reaction between molten Al and O_2_ when using a polymer gate	[119]
Al-CNTs@Cu_2_O	HE	At 520 °C, extrusion coefficient is 16:1	236.0	415.0	11.6	-	-	Strengthening contribution of Cu_2_O@CNT is 236 MPa	[138]
Al-Cu/CNT	SPS	600 °C (100 °C/min—10 min)	221.9	245.0	14.8	-	-	Improved wettability of the interface of composites and “Al-Cu-CNT” interlocking effect	[144]
Mg-2Al/CNT	HE/HP	500 °C, 180 s/550 °C, 2 h, 30 MPa	-	307.0	10.5	-	-	Exhibited strength–ductility synergy	[79]
Al-CNT@ SiO_2_ changed to Al–Al_4_O_4_C	SPS	610 °C	370.0	472.0	9.6	86.8	-	No degrading of ductility	[141]
Al-Cu/CNT	PM	300 r/min, 3 h, ball/powder mass ratio of 5:1	368.0	495.14	-	78.3	107.7	Density is 2.7 g·cm^3^ Compression strain/is 19.9%	[148]
7075Al-SiCp/0.5CNT	HP/HE	550 °C, 30 min, 300 MPa/400 °C, 1 h	504.6	575.9	5.4	101.3	191.1	The introduction of a hybrid SiCp reinforcing material (CNT) can help reduce the deposition of composites during aging	[111]
Al-Si-10Mg/1.5%CNT	SPS	540 °C, 18 min, 40 MPa	241.0	337.0	4.0	-	-	The uniform dispersion of 0.8 wt.% CNTs on the surface of the matrix improves the mechanical properties of the composites	[149]
Al_2_O_3_-ZrO_2_/CNT	Atmos-pheric plasma spraying	40 KW, 57.20 V	-	-	-	102.7	14.1 GPa	Plasticity index is 0.17 Shared stress is 332.0 MPa	[150]

PM^1^—Production method; SP^2^—Synthesis parameter; YS^3^—Yield strength, MPa; TS^4^—Tensile strength, MPa; E^5^—Elongation, %; YM^6^—Young’s module, GPa; HV^7^—Hardness values, HV; R^8^—Reference.

**Table 5:** Al–CNT composites and their electrical properties.

Composite	PM^1^	SP^2^	DD^3^	EC^4^	Comms	R^5^
Al-CNT	PM	Al:CNT is 95:5, 400 °C, 1 min	-	1.52·10^7^	No change in the hardness level, compared with Al	[68]
Al-4%CNTs/2GAg,NPs	SPS	580 °C, 50 MPa, 5 V, 300 amps	1.98·10^4^	4.21·10^5^	91.08 and 95.56% protection efficiency were obtained over that of the α-Al Matrix at Al-4%CNTs and Al-4%CNTs + 2%GAgNPs	[171]
Al-Cu/f-CNT	*EP	0.5 V, 5 min	-	72.93% for wire (coated by electrophoresis at 1.2 A)	Electrical resistivity is 3.29·10^−9^ Ωm	[175]

PM^1^—Production method; SP^2^—Synthesis parameter; DD^3^—Dislocation density; EC^4^—Electrical conductivity, S·m^−1^; R^5^—Reference; *EP—Electrophoresis.

**Table 6:** Al-CNT composites and their thermal properties.

Composite	PM^1^	SP^2^	TE^3^	TC^4^	Comms	R^5^
Al-0.5CNTs/L3	HP	150 rpm, 600 °C, 20 MPa, vacuum 10 Pa, 50 min	-	148.0	Molecular dynamics simulations revealed the heat transfer mechanism through the electron–phonon coupling effect	[145]
Al-Si/CNT	HE	550 °C, 1 mm/s, 16:1	16.8 × 10^−6^ (at 50–400 °C)	102.0	Yield strength is 235 MPa	[178]
Al-CNT	CVD/HE	550 °C, 30 min/400 °C, 16:1	-	246.6 (400 °C)	Ultimate strength is 186.2 MPa; minimum CTE is 25 × 10^−6^ K^−1^ (400 °C)	[179]
Al-3.7%CNT	3D printing	350 W, scan speed of 1250 mm/s, layerthickness of 30 mm and hatch spacing of 60 mm.	-	400.0	Detected the effect of the interface and thermal loading direction on the thermal conductivity of the composites	[180]
Al-Graphite Film/CNT	PM	570 °C, 50 MPa, 30 min	-	897.0	The in-plane TC improved due to the heat conduction pathway formed by CNTs on the interface	[181]
Al-5%CNT	3D RVE model	-	5.12 × 10^−6^ °C^−1^	n/a	The effect of increasing the volume fraction of CNTs leads to a remarkable decrease in the CTE of Al/CNT composites	[182]

PM^1^—Production method; SP^2^—Synthesis parameter; TE^3^—Thermal expansion, K^−1^; TC^4^—Thermal conductivity, W·m^−1^·K^−1^; R^5^—Reference.

## Data Availability

Not applicable.

## References

[1] Marques A., Guimarães B., Bartolomeu F., Miranda G., Silva F.S., Carvalho O. (2023). Multi-Material Inconel 718—Aluminium Parts Targeting Aerospace Applications: A Suitable Combination of Low-Weight and Thermal Properties. Opt. Laser Technol..

[2] Soni R., Verma R., Garg R.K., Singh H. (2024). Progress in Aerospace Materials and Ablation Resistant Coatings: A Focused Review. Opt. Laser Technol..

[3] Zheng X., Zhang H., Liu Z., Jiang R., Zhou X. (2022). Functional Composite Electromagnetic Shielding Materials for Aerospace, Electronics and Wearable Fields. Mater. Today Commun..

[4] Rajak D.K., Wagh P.H., Linul E. (2022). A Review on Synthetic Fibers for Polymer Matrix Composites: Performance, Failure Modes and Applications. Materials.

[5] Paladugu S.R.M., Sreekanth P.S.R., Sahu S.K., Naresh K., Karthick S.A., Venkateshwaran N., Ramoni M., Mensah R.A., Das O., Shanmugam R. (2022). A Comprehensive Review of Self-Healing Polymer, Metal, and Ceramic Matrix Composites and Their Modeling Aspects for Aerospace Applications. Materials.

[6] Sarmah P., Gupta K. (2024). Recent Advancements in Fabrication of Metal Matrix Composites: A Systematic Review. Materials.

[7] Fu W., Xue Y., Song X., Tian X., Wu G., Bian H., Hu S. (2024). Microstructural Evolution and Mechanical Properties of Al_2_O_3_/Al Joint Bonded by Bi_2_O_3_-B_2_O_3_-SiO_2_-ZnO-Al_2_O_3_ Glass. Ceram. Int..

[8] Feng X., Zhang M., Jiang T., Xie Y., Sun Z., Li W. (2024). Additive Friction Stir Deposition of an Al-Cu-Mg Alloy: Microstructure Evolution and Mechanical Properties. Mater. Charact..

[9] Verma P.K., Singh A., Kumar A., Malik N. (2025). Microstructure, Mechanical, and Wear Characteristics of Heat-Treated Aerospace-Grade Aluminium Composite Reinforced with HEA Particles. Mater. Chem. Phys..

[10] Prasad J., Sonwani R.K. (2024). Optimize Chemical Milling of Aluminium Alloys to Achieve Minimum Surface Roughness in Aerospace and Defense Industry. J. Indian Chem. Soc..

[11] Dursun T., Soutis C. (2014). Recent Developments in Advanced Aircraft Aluminium Alloys. Mater. Des. 1980–2015.

[12] Agarwal I. (2024). Composites, Metals, and Ceramics Used in the Boeing 787—A Material Overview. Int. J. Sci. Res. IJSR.

[13] Zhu L., Li N., Childs P.R.N. (2018). Light-Weighting in Aerospace Component and System Design. Propuls. Power Res..

[14] Vadivel M., Seenivasan S., Satishkumar P., Gomkale S., Saminathan R. (2024). Enhancement of Mechanical Properties in AA5083 Aluminum Metal Matrix Composites through Alumina and Titanium Carbide Reinforcement. Interactions.

[15] Muribwathoho O., Msomi V., Mabuwa S. (2024). An Analysis Comparing the Taguchi Method for Optimizing the Process Parameters of AA5083/Silicon Carbide and AA5083/Coal Composites That Are Fabricated via Friction Stir Processing. Appl. Sci..

[16] Krishnan B.R., Viravel D.K., Sundaram C.M., Ramkumaresh H. (2024). Wear Analysis of Al-SiC-Jute Ash Hybrid Composite via Stir Casting Process. Interactions.

[17] Lucas J.P., Stephens J.J., Greulich F.A. (1991). The Effect of Reinforcement Stability on Composition Redistribution in Cast Aluminum Metal Matrix Composites. Mater. Sci. Eng. A.

[18] Farid W., Li H., Wang Z., Cui H., Kong C., Yu H. (2024). Integrating Experimental and Computational Analyses for Mechanical Characterization of Titanium Carbide/Aluminum Metal Matrix Composites. Materials.

[19] Andilab B., Emadi P., Sydorenko M., Ravindran C. (2024). Influence of GNP Additions on the Microstructure, Mechanical Properties, and Electrical Conductivity of Cast A319 Aluminum Alloy. Int. J. Met..

[20] Zhu M., Shao Y., Zhao Y., Chua B.W., Du Z., Gan C.L. (2024). Interface Engineering in CF/Al Matrix Composites for Enhancement in Mechanical Strength and Anti-Corrosion Properties. Mater. Charact..

[21] Kundu S., Mondal S. (2024). Electro-Thermal and Mechanical Property Analysis of Powder Metallurgy Processed, Multi-Stage Ball Milled Aluminium-Copper-Multi Walled Carbon Nanotube Composite. Eng. Res. Express.

[22] Devadiga U., Fernandes P., Buradi A., Emma A.F. (2024). Significance of Addition of Carbon Nanotubes and Fly Ash on the Wear and Frictional Performance of Aluminum Metal Matrix Composites. Eng. Rep..

[23] Yao N., Lordi V. (1998). Young’s Modulus of Single-Walled Carbon Nanotubes. J. Appl. Phys..

[24] George R., Kashyap K.T., Rahul R., Yamdagni S. (2005). Strengthening in Carbon Nanotube/Aluminium (CNT/Al) Composites. Scr. Mater..

[25] Li L., Jiang H., Liu Y., Zhao Q., Tao J., Fan Y., Liu Y., Li C., Yi J. (2024). Improvement of Thermal Conductivity and Wear Property of Gr/EP Composites with CNTs/Cu Foam as 3-Dimensional Reinforcing Skeleton. J. Mater. Res. Technol..

[26] Kumar N., Soren S., Prasad R., Singh Y., Nautiyal H., Sharma A., Tiang S.S., Lim W.H. (2023). Optimization of Sintering Process Parameters by Taguchi Method for Developing Al-CNT-Reinforced Powder Composites. Crystals.

[27] Cao L., Chen B., Wan J., Kondoh K., Guo B., Shen J., Li J.S. (2022). Superior High-Temperature Tensile Properties of Aluminum Matrix Composites Reinforced with Carbon Nanotubes. Carbon.

[28] Adara P.P., Oyinbo S.T., Jen T.-C. (2024). Electrical and Mechanical Properties Variation of Al_2_O_3_–CaO-CNT(3,3) Nanomaterial Due to Al Vacancy and Temperature: DFT Approach. Results Mater..

[29] Yang K., Yang X., Liu E., Shi C., Ma L., He C., Li Q., Li J., Zhao N. (2017). Elevated Temperature Compressive Properties and Energy Absorption Response of In-Situ Grown CNT-Reinforced Al Composite Foams. Mater. Sci. Eng. A.

[30] Hussein M.A., Shahzad H.K., Patel F., Atieh M.A., Al-Aqeeli N., Baroud T.N., Laoui T. (2020). Porous Al_2_O_3_-CNT Nanocomposite Membrane Produced by Spark Plasma Sintering with Tailored Microstructure and Properties for Water Treatment. Nanomaterials.

[31] Kuzumaki T., Miyazawa K., Ichinose H., Ito K. (1998). Processing of Carbon Nanotube Reinforced Aluminum Composite. J. Mater. Res..

[32] Shivaramu H.T., Nayak U.V., Neelakantha V.L., Umashankar K.S., Boppana S.B., Ramachandra C.G., Kumar K.P., Ramesh S. (2024). Production of Al/MWCNT Nanocomposite by Powder Metallurgy to Enhance Dry Sliding Wear Performance Aided by Design of Experiment. Structural Composite Materials: Fabrication, Properties, Applications and Challenges.

[33] Deng C.F., Wang D.Z., Zhang X.X., Li A.B. (2007). Processing and Properties of Carbon Nanotubes Reinforced Aluminum Composites. Mater. Sci. Eng. A.

[34] Doğan K., Özgün M.İ., Sübütay H., Salur E., Eker Y., Kuntoğlu M., Aslan A., Gupta M.K., Acarer M. (2022). Dispersion Mechanism-Induced Variations in Microstructural and Mechanical Behavior of CNT-Reinforced Aluminum Nanocomposites. Arch. Civ. Mech. Eng..

[35] Ujah C., Popoola P., Popoola O., Aigbodion V. (2019). Enhanced Mechanical, Electrical and Corrosion Characteristics of Al-CNTs-Nb Composite Processed via Spark Plasma Sintering for Conductor Core. J. Compos. Mater..

[36] Ujah C.O., Popoola A.P.I., Popoola O.M., Aigbodion V.S. (2019). Enhanced Tribology, Thermal and Electrical Properties of Al-CNT Composite Processed via Spark Plasma Sintering for Transmission Conductor. J. Mater. Sci..

[37] Krishna A., Aravinda L.S., Murugan A., Kumar N.S., Sankar M.R., Nagahanumaiah, Reddy K.N., Balashanmugam N. (2022). A Study on Wafer Scalable, Industrially Applicable CNT Based Nanocomposites of Al-CNT, Cu-CNT, Ti-CNT, and Ni-CNT as Thermal Interface Materials Synthesised by Thin Film Techniques. Surf. Coat. Technol..

[38] Li X., Zhang Z., Peng Y., Yan D., Tan Z., Zhou Q., Wang K., Zhou M. (2022). Microstructure and Mechanical Properties of Underwater Friction Stir Welding of CNT/Al-Cu-Mg Composites. J. Mater. Res. Technol..

[39] Soni S., Thomas B., Kar V. (2020). A Comprehensive Review on CNTs and CNT-Reinforced Composites: Syntheses, Characteristics and Applications. Mater. Today Commun..

[40] Zuo G., Bai Y., Shi S., Tan Z., Fan W., Li Z., Hao H. (2024). Interfacial Healing Behavior of CNTs/Al Composites in Solid-State Additive Forging. J. Manuf. Process..

[41] Akbarpour M.R., Pouresmaeil A. (2018). The Influence of CNTs on the Microstructure and Strength of Al-CNT Composites Produced by Flake Powder Metallurgy and Hot Pressing Method. Diam. Relat. Mater..

[42] Li P.Y., Li X.N., Dong Z.Y., Liu Z.Y., Chen L.Q., Xiao B.L., Ma Z.Y. (2023). Microstructure Evolution and Strength-Ductility Improving Mechanism Change of Heterogeneous CNT/2009Al Composite after Hot Rolling. J. Alloys Compd..

[43] bin Ariffin M.A., bin Muhamad M.R., Raja S., Jamaludin M.F., Yusof F., Suga T., Liu H., Morisada Y., Fujii H. (2022). Friction Stir Alloying of AZ61 and Mild Steel with Cu-CNT Additive. J. Mater. Res. Technol..

[44] Zuo G., Bai Y., Tan Z., Fan W., Shi S., Hao H. (2024). Temperature Effects on Solid State Bonding Joints of Ultrafine-Grained CNT/Al–Cu–Mg Composites. Compos. Part B Eng..

[45] Yuan C., Zhang Z., Tan Z., Xu L., Zhang S., Fan G., Zhang P., Li Z. (2021). Enhanced Ductility by Mg Addition in the CNT/Al-Cu Composites via Flake Powder Metallurgy. Mater. Today Commun..

[46] Cao L., Chen B., Wan J., Shen J., Li S., Liu S., Li J. (2023). Unraveling the Dispersion Mechanism of Carbon Nanotubes in Aluminum Powder Particles during High Energy Ball Milling by FIB-TEM Study. Powder Technol..

[47] Wang T., Xu F., Sun L., Miao L., Liao L., Wei S., Yin Q., Zhang K., Li Y., Wu Y. (2021). Improved Performance of Hydrogen Generation for Al–Bi-CNTs Composite by Spark Plasma Sintering. J. Alloys Compd..

[48] Ramachandran K., Gnanasagaran C.L., Pazhani A., Kumar V.H., Xavior M.A., Subramani R.R., Arunkumar T. (2023). Impact Behaviour of MWCNTs Reinforced YSZ and Al_2_O_3_ Ceramic-Nanocomposites Prepared via Vacuum Hot-Pressing Technique. J. Mater. Res. Technol..

[49] Ma K., Liu Z.Y., Liu B.S., Xiao B.L., Ma Z.Y. (2021). Improving Ductility of Bimodal Carbon Nanotube/2009Al Composites by Optimizing Coarse Grain Microstructure via Hot Extrusion. Compos. Part Appl. Sci. Manuf..

[50] Dhore V.G., Rathod W.S., Patil K.N. (2018). Investigation of Mechanical Properties of Carbon Nanotubes Reinforced Aluminium Composite by Metal Injection Molding. Mater. Today Proc..

[51] Zhong K., Zhou J., Zhao C., Yun K., Qi L. (2022). Effect of Interfacial Transition Layer with CNTs on Fracture Toughness and Failure Mode of Carbon Fiber Reinforced Aluminum Matrix Composites. Compos. Part Appl. Sci. Manuf..

[52] Guan H.D., Li C.J., Peng Y.Z., Gao P., Feng Z.X., Liu Y.C., Li J.N., Tao J.M., Yi J.H. (2022). Fe-Based Metallic Glass Particles Carry Carbon Nanotubes to Reinforce Al Matrix Composites. Mater. Charact..

[53] Liu L., Li S., Zhang X., Pan D., Gao L., Chen B., Umeda J., Kondoh K. (2021). Syntheses, Microstructure Evolution and Performance of Strength-Ductility Matched Aluminum Matrix Composites Reinforced by Nano SiC-Cladded CNTs. Mater. Sci. Eng. A.

[54] Saba F., Chen M., Ziaei H., Fan G., Tan Z., Li Z. (2023). Fabrication of High-Content Hybrid-Reinforced Al Nanocomposites by in-Situ Reaction of TiO_2_-Decorated CNTs and Matrix. Diam. Relat. Mater..

[55] Geng H., Chen B., Cao L., Wan J., Shen J., Kondoh K., Li J. (2024). Aging Behavior, Microstructure and Mechanical Properties of Al-Cu-Mg Alloy Matrix Composites Reinforced with Carbon Nanotubes. Mater. Sci. Eng. A.

[56] Zhang Z., Xiao Y., Xu J., He M., Luo Y., Xiang J. (2022). Understanding the Influencing Mechanism of CNTs on the Microstructure and Mechanical Properties of Semi-Solid Stir Casting Al-Cu-Mg Alloys. J. Mater. Res. Technol..

[57] Zhu W., Liu H., Xing S., Jiang C., Ji V. (2024). Surface Mechanical Property and Residual Stress Stability of Nanostructured CNT/Al-Cu-Mg Composites Induced by Shot Peening. Mater. Charact..

[58] Sasani F., Taheri A.K., Pouranvari M. (2022). Correlation between Microstructure and Mechanical Properties of AlMg6/CNT-Al Composite Produced by Accumulative Roll Bonding Process: Experimental and Modelling Analysis. Mater. Sci. Eng. A.

[59] Zhang B., Huang Y., Dou Z., Wang J., Huang Z. (2024). Refractory High-Entropy Alloys Fabricated by Powder Metallurgy: Progress, Challenges and Opportunities. J. Sci. Adv. Mater. Devices.

[60] Wei Y., Luo L.-M., Liu H.-B., Zan X., Song J.-P., Xu Q., Zhu X.-Y., Wu Y.-C. (2020). A Powder Metallurgy Route to Fabricate CNT-Reinforced Molybdenum-Hafnium-Carbon Composites. Mater. Des..

[61] Fan G., Xu R., Tan Z., Zhang D., Li Z. (2014). Development of Flake Powder Metallurgy in Fabricating Metal Matrix Composites: A Review. Acta Metall. Sin. Engl. Lett..

[62] Xu R., Tan Z., Xiong D., Fan G., Guo Q., Zhang J., Su Y., Li Z., Zhang D. (2017). Balanced Strength and Ductility in CNT/Al Composites Achieved by Flake Powder Metallurgy via Shift-Speed Ball Milling. Compos. Part Appl. Sci. Manuf..

[63] Sattari S., Jahani M., Atrian A. (2017). Effect of Volume Fraction of Reinforcement and Milling Time on Physical and Mechanical Properties of Al7075–SiC Composites Fabricated by Powder Metallurgy Method. Powder Metall. Met. Ceram..

[64] Liu Z.Y., Zhao K., Xiao B.L., Wang W.G., Ma Z.Y. (2016). Fabrication of CNT/Al Composites with Low Damage to CNTs by a Novel Solution-Assisted Wet Mixing Combined with Powder Metallurgy Processing. Mater. Des..

[65] Choi H., Shin J., Min B., Park J., Bae D. (2009). Reinforcing Effects of Carbon Nanotubes in Structural Aluminum Matrix Nanocomposites. J. Mater. Res..

[66] Peng T., Chang I. (2015). Uniformly Dispersion of Carbon Nanotube in Aluminum Powders by Wet Shake-Mixing Approach. Powder Technol..

[67] Sadeghi B., Cavaliere P., Perrone A., Castro M.M. (2024). Optimizing Ball Milling Parameters for Controlling the Internal Microstructure and Tensile Characteristics of a Laminated Carbon Nanotube/Aluminum–Copper–Magnesium Composite. Results Mater..

[68] Kim D., Hirayama Y., Liu Z., Takagi K., Kobashi M. (2023). Fabrication of Al-CNT Composite with High Hardness and Electrical Conductivity by Controlling Al_4_C_3_ Formation. J. Alloys Compd..

[69] Ardila-Rodríguez L.A., Menezes B.R.C., Pereira L.A., Oliveira A.C., Travessa D.N. (2019). Titanium Dioxide Protection against Al4C3 Formation during Fabrication of Aluminum-TiO_2_ Coated MWCNT Composite. J. Alloys Compd..

[70] Toozandehjani M., Ostovan F., Jamaludin K.R., Amrin A., Matori K.A., Shafiei E. (2020). Process−microstructure−properties Relationship in Al−CNTs−Al_2_O_3_ Nanocomposites Manufactured by Hybrid Powder Metallurgy and Microwave Sintering Process. Trans. Nonferrous Met. Soc. China.

[71] Zhang X., Li X., Liu L., Li B., Hou X., Pan D., Gao L., Li S. (2023). Interface Regulation Strategy of Al-CNTs Composite Induced by Al-Si Eutectic Reaction and Its Strengthening Mechanism. J. Mater. Sci. Technol..

[72] Wan J., Chen B., Feng D., Cao L., Shen J., Guo B., Li J.S. (2022). Strengthening Efficiency Competition between Carbon Nanotubes (CNTs) and in-Situ Al_4_C_3_ Nanorods in CNTs/Al Composites Influenced by Alumina Characteristics. Compos. Part Appl. Sci. Manuf..

[73] Peng T., Chang I. (2014). Mechanical Alloying of Multi-Walled Carbon Nanotubes Reinforced Aluminum Composite Powder. Powder Technol..

[74] Payandehpeyman J., Hedayatian M., Mazaheri M. (2024). Predicting Effective Elastic Modulus of CNT Metal Matrix Nanocomposites: A Developed Micromechanical Model with Agglomeration and Interphase Effects. Compos. Struct..

[75] Yi C., Chen X., Gou F., Dmuchowski C.M., Sharma A., Park C., Ke C. (2017). Direct Measurements of the Mechanical Strength of Carbon Nanotube—Aluminum Interfaces. Carbon.

[76] Awotunde M.A., Olubambi P.A., Chen D. (2022). Compressive Deformation Behaviour and Toughening Mechanisms of Spark Plasma Sintered NiAl-CNT Composites. Ceram. Int..

[77] Carneiro Í., Viana F., Vieira M., Fernandes J., Simões S. (2019). EBSD Analysis of Metal Matrix Nanocomposite Microstructure Produced by Powder Metallurgy. Nanomaterials.

[78] Liu Y., Ning Y., Zekun Y., Li H., Miao X., Li Y., Zhao Z. (2016). Plastic Deformation and Dynamic Recrystallization of a Powder Metallurgical Nickel-Based Superalloy. J. Alloys Compd..

[79] Li B., Chen H., Li G., Wei G., Xie W. (2023). Strengthening and Toughening Mechanisms of CNTs/Mg–Al Composites Prepared via Powder Metallurgy Combined with Hot Extrusion. Vacuum.

[80] Wang S., Yuan T., Liu L., Wang L., Jiang X., Shan H., Chen S., Zhao P. (2024). Microstructure and Strengthening Mechanism of TIG Welded Joint of AZ31 Alloy Based on FSP Technique. J. Manuf. Process..

[81] Sharma A., Fujii H., Paul J. (2020). Influence of Reinforcement Incorporation Approach on Mechanical and Tribological Properties of AA6061-CNT Nanocomposite Fabricated via FSP. J. Manuf. Process..

[82] Jain V.K.S., Yazar K.U., Muthukumaran S. (2019). Development and Characterization of Al5083-CNTs/SiC Composites via Friction Stir Processing. J. Alloys Compd..

[83] Izadi H., Gerlich A.P. (2012). Distribution and Stability of Carbon Nanotubes during Multi-Pass Friction Stir Processing of Carbon Nanotube/Aluminum Composites. Carbon.

[84] Parikh V.K., Badgujar A.D., Ghetiya N.D. (2022). Effect of Friction Stir Processing Parameters on Microstructure and Microhardness of Aluminium Based Metal Matrix Composites. Mater. Today Proc..

[85] Khan M., Rehman A., Aziz T., Naveed K., Ahmad I., Subhani T. (2017). Cold Formability of Friction Stir Processed Aluminum Composites Containing Carbon Nanotubes and Boron Carbide Particles. Mater. Sci. Eng. A.

[86] Pragada V., Soni V., Ghetiya N.D., Bharti S. (2022). Influence of Tool Travel Direction on Microhardness and Tribological Properties of AA2014/SiC-CNT Hybrid Surface Composites Produced by Multi-Pass Friction Stir Processing. Mater. Today Proc..

[87] Dinesh Kumar D., Balamurugan A., Suresh K.C., Suresh Kumar R., Jayanthi N., Ramakrishnan T., Hasane Ahammad S.K., Mayakannan S., Venkatesa Prabhu S. (2023). Study of Microstructure and Wear Resistance of AA5052/B4C Nanocomposites as a Function of Volume Fraction Reinforcement to Particle Size Ratio by ANN. J. Chem..

[88] Zhang S., Wang T., Jiang Z. (2023). Carbon Nanotubes/Aluminum Interface Structure and Its Effects on the Strength and Electrical Conductivity of Aluminum. J. Mater. Res. Technol..

[89] Liu Z.Y., Xiao B.L., Wang W.G., Ma Z.Y. (2013). Developing High-Performance Aluminum Matrix Composites with Directionally Aligned Carbon Nanotubes by Combining Friction Stir Processing and Subsequent Rolling. Carbon.

[90] Li X., Zhang Z., Peng Y., Yan D., Tan Z., Zhou Q., Wang K. (2021). In Situ Synthesized Nano-Al_4_C_3_ Reinforced Aluminum Matrix Composites via Friction Stir Processing. J. Mater. Res. Technol..

[91] Du Z., Tan M.J., Guo J.F., Bi G., Wei J. (2016). Fabrication of a New Al-Al2O3-CNTs Composite Using Friction Stir Processing (FSP). Mater. Sci. Eng. A.

[92] Kumar R., Singh J., Sharma S., Li C., Królczyk G., Wojciechowski S. (2022). Neutrosophic Entropy-Based Ingenious Measurement for Fast Fourier Transforms Based Classification of Process-Parameters and Wear Resistance of Friction-Stir Processed Hybrid AA7075-B4C Aluminium Metal-Matrix Composites. J. Mater. Res. Technol..

[93] Pasha S.A., Reddy P.R., Laxmi Narayana P. (2017). Wear Behavior and Microstructural Characterization of AA7075/MWCNT Surface Composites Fabricated through Friction Stir Processing. IOSR J. Mech. Civ. Eng..

[94] Golla C.B., Babar Pasha M., Rao R.N., Ismail S., Gupta M. (2023). Influence of TiC Particles on Mechanical and Tribological Characteristics of Advanced Aluminium Matrix Composites Fabricated through Ultrasonic-Assisted Stir Casting. Crystals.

[95] Ekhlasiosgouei O., Ebrahimi R., Hasheminiasari M., Molin S. (2023). An Investigation of Microstructural Basis for Corrosion Behavior of Al-CNT Composites Fabricated by SPS. Diam. Relat. Mater..

[96] Liao Z., He Q., Zhang W., Zhang F., Wang W., Fu Z. (2024). B_4_C Ceramics with Increased Dislocation Density Fabricated by Reactive Spark Plasma Sintering via Carbon Nanotubes–Boron Mixture. Ceram. Int..

[97] Tsukamoto H. (2023). Chemical and Mechanical Treatments for Enhancement of Carbon Nanotube Reinforced Aluminum Matrix Composites. Mater. Sci. Eng. A.

[98] Chen D., Chen G., Deng M., Wang H., Huang Z., Qi J., Lu T. (2022). Fabrication and Mechanical Properties of Multi-Walled Carbon Nanotubes Doped AlN Ceramics Prepared by Spark Plasma Sintering. Ceram. Int..

[99] Heydari S., Sajjadi S.A., Babakhani A., Eskandari H., Nateq B. (2023). An Investigation on the Effect of Al_4_C_3_ on Microstructure and Mechanical Properties of Carbon Nanotube Reinforced Aluminum Composite. Ceram. Int..

[100] Suslova E., Savilov S., Egorov A., Shumyantsev A., Lunin V. (2019). Carbon Nanotube Frameworks by Spark Plasma Sintering. Microporous Mesoporous Mater..

[101] Wan J. (2024). CNT-Induced Heterogeneous Matrix Grain Structure in CNTs/Al Composites. Carbon.

[102] Huang L., Liu B., Wang Z., Yuan J. (2023). Effect of Carbon Nanotube Content on the Microstructure and Mechanical Properties of CNTs/TiAl Alloys. Ceram. Int..

[103] Trinh P.V., Lee J., Kang B., Minh P.N., Phuong D.D., Hong S.H. (2022). Mechanical and Wear Properties of SiCp/CNT/Al6061 Hybrid Metal Matrix Composites. Diam. Relat. Mater..

[104] Yang C.M.Y., Li X., Li C.J., Peng Y.Z., Xing Y., Feng Z.X., Tan J., Tao J.M., Li Z.L., Wang Y.R. (2023). Interface and Strengthening Mechanisms of Al Matrix Composites Reinforced with In-Situ CNTs Grown on Ti Particles. Mater. Des..

[105] Guo B., Ni S., Yi J., Shen R., Tang Z., Du Y., Song M. (2017). Microstructures and Mechanical Properties of Carbon Nanotubes Reinforced Pure Aluminum Composites Synthesized by Spark Plasma Sintering and Hot Rolling. Mater. Sci. Eng. A.

[106] Jagannatham M., Sankaran S., Haridoss P. (2015). Microstructure and Mechanical Behavior of Copper Coated Multiwall Carbon Nanotubes Reinforced Aluminum Composites. Mater. Sci. Eng. A.

[107] Yu M., Kim M., Yoon B., Oh S., Nam D.-H., Kwon H. (2014). Carbon Nanotubes/Aluminum Composite as a Hydrogen Source for PEMFC. Int. J. Hydrogen Energy.

[108] Zhou X., Liu Y., Wang Z., Li H. (2022). Microstructure and Mechanical Properties of Aluminum Matrix Composites Reinforced by One-Dimensional/Two-Dimensional Hybrid Carbon Nanophases. Chin. J. Nonferrous Met..

[109] Yang S., Luo H., Wang L., Guang Z., Zhang P., Liu Y. (2023). Interface Structure and Bonding Strength of Metallurgical Bonded Aluminum Foam Sandwich (AFS) Fabricated by Hot-Pressing. Vacuum.

[110] Chang J.-M., Yadav B.N., Mandal A., Tiwari J.K., Kam K.-H., Liu D.-S., Lin P.C. (2024). Carbon Nanotubes Used to Enhance the Wear Properties of AlSi10Mg/CNTs Nanocomposites Prepared through Additive Manufacturing. Diam. Relat. Mater..

[111] Wang X. (2023). Effect of the CNTs into SiCp-Al Interfacial Micro-Zones on Ageing Precipitation Behavior, Microstructure and Mechanical Properties of SiCp(CNT)/Al–Zn–Mg–Cu Composites. Compos. Part B.

[112] Wang X., Wang S., Wang X., Su Y., Yue Z., Cao H., Zhang D., Ouyang Q. (2024). Synergistic Strengthening-Toughening Effect of SiCp(CNT) Hybrid Reinforcements on Mg-Compensated SiCp(CNT)/Al–Zn–Mg–Cu Composites. Mater. Sci. Eng. A.

[113] Xiang J., Zheng Y., Li J., Tan Z. (2023). Mechanical Response of CNT/2024Al Composite to Compression and Tension at Different Strain Rates. Metals.

[114] Sha J., Li J., Wang S., Zhang Z., Wang Y., Dai J. (2016). Microstructure and Mechanical Properties of Hot-Pressed ZrC–Ti–CNTs Composites. Mater. Des..

[115] Guo B., Song M., Yi J., Ni S., Shen T., Du Y. (2017). Improving the Mechanical Properties of Carbon Nanotubes Reinforced Pure Aluminum Matrix Composites by Achieving Non-Equilibrium Interface. Mater. Des..

[116] Yildirim M., Özyürek D., Gürü M. (2016). Investigation of Microstructure and Wear Behaviors of al Matrix Composites Reinforced by Carbon Nanotube. Fuller. Nanotub. Carbon Nanostruct..

[117] Aborkin A.V., Elkin A.I., Reshetniak V.V., Ob’edkov A.M., Sytschev A.E., Leontiev V.G., Titov D.D., Alymov M.I. (2021). Thermal Expansion of Aluminum Matrix Composites Reinforced by Carbon Nanotubes with In-Situ and Ex-Situ Designed Interfaces Ceramics Layers. J. Alloys Compd..

[118] Li Y., Li J., Jin Q., Li Z., Li L., Sun Y. (2024). Study on the Mechanism of CNTs Regulating the Microstructures and Properties of Al–Cu–Mg Alloy. Crystals.

[119] Oh S.-H., Kang H.-J., Yoon P.-H., Lee G.-H., Shin S.-M., Choi Y.-S., Park J.-Y. (2023). Feasibility Study on the Fabricating of Carbon-Nanotube-Reinforced Al-Si-Cu Alloy Matrix Composites Using Oxygen-Replacing Die Casting Process. Metals.

[120] Popov V.V., Pismenny A., Larianovsky N., Lapteva A., Safranchik D. (2021). Corrosion Resistance of Al–CNT Metal Matrix Composites. Materials.

[121] Usef A.P., Bhajantri V., Kannoth V., Jambagi S.C. (2021). Influence of Carbon Nanotube Reinforcement on the Heat Transfer Coefficient, Microstructure, and Mechanical Properties of a Die Cast Al-7Si-0.35Mg Alloy. J. Alloys Compd..

[122] Zhang J., Cinkilic E., Huang X., Wang G.G., Liu Y.C., Weiler J.P., Luo A.A. (2023). Optimization of T5 Heat Treatment in High Pressure Die Casting of Al–Si–Mg–Mn Alloys by Using an Improved Kampmann-Wagner Numerical (KWN) Model. Mater. Sci. Eng. A.

[123] Maniraj S., Anand K., Anbarasu R., Aravindan A.A., Gokul G., Logendran R. (2022). Impacts of Carbon Nano Tubes (CNT) and Boron Carbide (B4C) Particles on Material Properties of al 6061. Mater. Today Proc..

[124] Larianovsky N., Popov V., Katz-Demyanetz A., Fleisher A., Meyers D.E., Chaudhuri R.S. (2019). Production of Al Metal Matrix Composites Reinforced With Carbon Nanotubes by Two-Stage Melt-Based HPDC-CE Method. J. Eng. Mater. Technol..

[125] Sarkar S., Ray S., Dey U., Kumar C., Chakraborti P.C., Mukhopadhyay G., Kumar C.S., Roy S. (2023). Establishment of a Facile Technique to Fabricate Bulk AA6061/CNT Composite with Improved Mechanical Properties Using Combined Stir-Casting and Squeeze-Casting. Mater. Today Commun..

[126] Li Q., Rottmair C.A., Singer R.F. (2010). CNT Reinforced Light Metal Composites Produced by Melt Stirring and by High Pressure Die Casting. Compos. Sci. Technol..

[127] Morovvati M.R., Mollaei-Dariani B., Lalehpour A., Toghraie D. (2023). Fabrication and Finite Element Simulation of Aluminum/Carbon Nanotubes Sheet Reinforced with Thermal Chemical Vapor Deposition (TCVD). J. Mater. Res. Technol..

[128] Zhang Y., Wang Q., Chen G., Ramachandran C.S. (2020). Mechanical, Tribological and Corrosion Physiognomies of CNT-Al Metal Matrix Composite (MMC) Coatings Deposited by Cold Gas Dynamic Spray (CGDS) Process. Surf. Coat. Technol..

[129] Liu X., Li J., Liu E., Li Q., He C., Shi C., Zhao N. (2018). Effectively Reinforced Load Transfer and Fracture Elongation by Forming Al4C3 for In-Situ Synthesizing Carbon Nanotube Reinforced Al Matrix Composites. Mater. Sci. Eng. A.

[130] Tang J., Fan G., Li Z., Li X., Xu R., Li Y., Zhang D., Moon W.-J., Kaloshkin S.D., Churyukanova M. (2013). Synthesis of Carbon Nanotube/Aluminium Composite Powders by Polymer Pyrolysis Chemical Vapor Deposition. Carbon.

[131] Liu Y., Li S., Misra R.D.K., Geng K., Yang Y. (2020). Planting Carbon Nanotubes within Ti-6Al-4V to Make High-Quality Composite Powders for 3D Printing High-Performance Ti-6Al-4V Matrix Composites. Scr. Mater..

[132] Huang Y., Su Y., Guo X., Guo Q., Ouyang Q., Zhang G., Zhang D. (2017). Fabrication and Thermal Conductivity of Copper Coated Graphite Film/Aluminum Composites for Effective Thermal Management. J. Alloys Compd..

[133] Meng L., Wang X., Ning J., Hu X., Fan G., Wu K. (2018). Beyond the Dimensional Limitation in Bio-Inspired Composite: Insertion of Carbon Nanotubes Induced Laminated Cu Composite and the Simultaneously Enhanced Strength and Toughness. Carbon.

[134] Zhang W., Zhao H., Hu X., Ju D. (2021). A Novel Processing for CNT-Reinforced Mg-Matrix Laminated Composites to Enhance the Electromagnetic Shielding Property. Coatings.

[135] Bhat A., Balla V.K., Bysakh S., Basu D., Bose S., Bandyopadhyay A. (2011). Carbon Nanotube Reinforced Cu–10Sn Alloy Composites: Mechanical and Thermal Properties. Mater. Sci. Eng. A.

[136] Eren O., Kamara A.M., Sezer H.K., Marimuthu S. (2024). Synthesis of Aluminium Nitride-Based Coatings on Mild Steel Substrates Utilising an Integrated Laser/Sol–Gel Method. Photonics.

[137] Shen H.-Z., Wang Y., Chen S.-M., Shen P. (2021). Wettability and Reactivity between Molten Aluminum and Randomly Aligned Carbon Nanotubes. J. Mater. Sci..

[138] Rong X., Chen X., Zhao D., Zhang X., He C., Shi C., Zhao N. (2023). High Mechanical Strengthened CNTs/Al Composite Concepts with Robust Interface and Intragranular Reinforcement Achieved via Interfacial Thermite Reaction. Compos. Part Appl. Sci. Manuf..

[139] Qiu C., Su Y., Yang J., Wang X., Chen B., Ouyang Q., Zhang D. (2021). Microstructural Characteristics and Mechanical Behavior of SiC(CNT)/Al Multiphase Interfacial Micro-Zones via Molecular Dynamics Simulations. Compos. Part B Eng..

[140] Herzallah H., Elsayd A., Shash A., Adly M. (2020). Effect of Carbon Nanotubes (CNTs) and Silicon Carbide (SiC) on Mechanical Properties of Pure Al Manufactured by Powder Metallurgy. J. Mater. Res. Technol..

[141] Ziaei H., Fan G., Tan Z., Zhang Y., Zhao L., Li Z., Li Z. (2024). SiO_2_ Coating on CNTs to Fabricate the Al4O4C-Al Composite with Superior Young’s Modulus. Mater. Charact..

[142] Ramesh R., Thirugnanasambantham K.G., Ravi L., Sivakumar P., Giridhar D. (2024). Effect of Agglomeration in Carbon Nanotube (CNT) Reinforced Aluminum (Al) Composites: A Review. AIP Conf. Proc..

[143] Wang M., Li Y., Chen B., Shi D., Umeda J., Kondoh K., Shen J. (2021). The Rate-Dependent Mechanical Behavior of CNT-Reinforced Aluminum Matrix Composites under Tensile Loading. Mater. Sci. Eng. A.

[144] Chen J., Yan L., Liang S., Cui X., Liu C., Wang B., Zou L. (2022). Remarkable Improvement of Mechanical Properties of Layered CNTs/Al Composites with Cu Decorated on CNTs. J. Alloys Compd..

[145] Shen M., Hao Z., Song J., An M., Ying T., Xue X., Gao Y., Yang Z. (2024). Architectural and Component Design of CNTs/Al Hierarchical Composite for Enhanced Mechanical/Thermal Properties. J. Mater. Res. Technol..

[146] Chowdhury H., Masud A. (2016). Stress Distribution in CNT-Aluminum Matrix Composite by Changing Distances between CNT Bundles. J. Nav. Archit. Mar. Eng..

[147] Carneiro Í., Simões S. (2022). Investigation of Mechanical Properties of Al/CNT Nanocomposites Produced by Powder Metallurgy. Appl. Sci..

[148] Luo S., Wu Y., Chen B., Song M., Yi J., Guo B., Wang Q., Yang Y., Li W., Yu Z. (2022). Effects of Cu Content on Microstructures and Compressive Mechanical Properties of CNTs/Al-Cu Composites. Trans. Nonferrous Met. Soc. China.

[149] Pan J., Zou L., Liao Z., Lin Z., Chen J. (2023). Study on the Properties of Carbon Nanotube (CNTs) Reinforced AlSi10Mg Composites Fabricated by Powder Metallurgy. Materials.

[150] Kushwaha S., Bhadauria A., Bajpai S., Tiwari A., Pandey K.K., Keshri A.K., Balani K. (2023). Mechanical, Microstructural, and Fretting Wear Behaviour of Al_2_O_3_–ZrO_2_-CNT Based Bimodal Composite Coatings. Wear.

[151] Abdeltawab N.M., Esawi A.M.K., Wifi A. (2023). Investigation of the Wear Behavior of Dual-Matrix Aluminum–(Aluminum–Carbon Nanotube) Composites. Metals.

[152] Zhu W., Liu H., Xing S., Jiang C., Ji V. (2024). Effect of Dual Shot Peening on Microstructure and Wear Performance of CNT/Al-Cu-Mg Composites. Materials.

[153] Wang L., Zhang Z., Luo Y., Xiao Y., Tan F., Liu K. (2022). Understanding the Influencing Mechanism of CNTs on the Microstructures and Wear Characterization of Semi-Solid Stir Casting Al-Cu-Mg-Si Alloys. Metals.

[154] Wang L., Liu Y., Wu J., Zhang X. (2017). Mechanical Properties and Friction Behaviors of CNT/AlSi10Mg Composites Produced by Spark Plasma Sintering. Int. J. Miner. Met. Mater..

[155] Sarkar S., Dam B., Dey U., Mandal N., Kumar C.S., Manna I., Roy S. (2025). Effect of Dispersion Technique and Applied Load on the Dry Sliding Wear Behavior of Combined Stir-Squeeze-Cast AA6061–0.5 Wt % CNT Composite against a Steel Counter Body at Both Room Temperature and Elevated Temperature. Wear.

[156] Sharma R., Sasikumar C., Baral J. (2024). Wear Behaviour of Al-MWCNT Composites by Varying MWCNTs Concentration. J. Alloys Metall. Syst..

[157] Hosseini S., Novák P., Alishahi M., Kačenka Z., Šittner P. (2024). Trade-Off Between Wear/Corrosion Performance and Mechanical Properties in D-AlNiCo Poly-Quasicrystals Through CNT Addition to the Microstructure. Metals.

[158] Al-Ashwan Z.H., Hayat U., Toor I.H., Hassan S.F., Saheb N. (2020). Corrosion Behavior of Spark Plasma Sintered Alumina and Al_2_O_3_-SiC-CNT Hybrid Nanocomposite. Mater. Res..

[159] Arora G.S., Saxena K.K., Mohammed K.A., Prakash C., Dixit S. (2022). Manufacturing Techniques for Mg-Based Metal Matrix Composite with Different Reinforcements. Crystals.

[160] Yan H., Li L., Hu H., Huang W. (2024). Influence of Al2O3 and H-BN on Wear and Corrosion Performance of IN625 Nickel-Based Coating. Coatings.

[161] Li N., Liu W., Wang Y., Zhao Z., Yan T., Zhang G., Xiong H. (2021). Laser Additive Manufacturing on Metal Matrix Composites: A Review. Chin. J. Mech. Eng..

[162] Chen Y., Shen J., Hu S., Zhen Y., Zhao H. (2024). Corrosion Behavior of CMT Cladding Layer of AZ91 Magnesium Alloy Subjected to Friction Stir Processing. Materials.

[163] Ananiadis E.A., Karantzalis A.E., Sfikas A.K., Georgatis E., Matikas T.E. (2023). Aluminium Matrix Composites Reinforced with AlCrFeMnNi HEA Particulates: Microstructure, Mechanical and Corrosion Properties. Materials.

[164] Ammisetti D.K., Kruthiventi S.S.H., Vinjavarapu S., Babu N.N., Gandepudi J.R., Battula S.K. (2024). A Review on Reinforcements, Fabrication Methods, and Mechanical and Wear Properties of Titanium Metal Matrix Composites. J. Eng. Appl. Sci..

[165] Singh G., Ablyaz T.R., Shlykov E.S., Muratov K.R., Bhui A.S., Sidhu S.S. (2020). Enhancing Corrosion and Wear Resistance of Ti6Al4V Alloy Using CNTs Mixed Electro-Discharge Process. Micromachines.

[166] Wei Z. (2023). Micro-Electrolysis Based Nitrate Reduction from Aqueous Solution by CNTs-Al-Cu Composite under Alkaline Environment. Chemosphere.

[167] Kumar G., Jyotheender K., Srivastava C. (2022). Effect of Grain Boundary Constitution on Corrosion Behaviour in Cobalt-Carbon Nanotube Composite Coatings. Materialia.

[168] Burleigh T. (2003). Corrosion of Aluminum and Its Alloys. Shreir’s Corrosion.

[169] Okokpujie I.P., Nakpoberuo D.O., Emojeya D.O., Azeez T.M., Tartibu L.K. (2024). Investigating Corrosion and Surface Hardness of Al6061 in Machining Fluids with Variable CNT Concentrations. Rev. Compos. Matér. Avancés.

[170] Ujah C., Popoola P., Popoola O., Aigbodion V.S. (2020). Influence of CNTs Addition on the Mechanical, Microstructural, and Corrosion Properties of Al Alloy Using Spark Plasma Sintering Technique. Int. J. Adv. Manuf. Technol..

[171] Aigbodion V.S. (2021). Microstructural Evolution, Electrical Conductivity, and Electrochemical Analysis of α-Al-CNTs-GAg.NPs High-Conductor Nanocomposite. Chem. Data Collect..

[172] Rondineau A., Gaillet L., Dieng L., Langlois S. (2022). Degradation of Steel Wires in Bimetallic Aluminum–Steel Conductors Exposed to Severe Corrosion Conditions. Corros. Mater. Degrad..

[173] Alraddadi S., Assaedi H. (2024). Phase Evolution, Mechanical, and Electrical Properties of Lightweight Ceramic Prepared Using Scoria at Low Temperature. Crystals.

[174] Devesa S., Amorim C.O., Belo J.H., Araújo J.P., Teixeira S.S., Graça M.P.F., Costa L.C. (2024). Comprehensive Characterization of Bi_1.34_Fe_0.66_Nb_1.34_O_6.35_ Ceramics: Structural, Morphological, Electrical, and Magnetic Properties. Magnetochemistry.

[175] Rodrigues F., Pinheiro P., Sousa M., Angélica R., Paz S., Reis M. (2022). Electrical Properties of Iodine-Doped Cu/f-CNT Coated Aluminum Wires by Electrophoresis with Copper Sulfate Solution. Metals.

[176] Zhou W., Zhou Z., Kubota K., Ono H., Nomura N., Kawasaki A. (2020). Design of High-Performance Al4C3/Al Matrix Composites for Electric Conductor. Mater. Sci. Eng. A.

[177] Jin Y., Wei S., Yang Z., Cui C., Wang J., Li D., Qian W. (2024). Li-Ion Batteries with a Binder-Free Cathode of Carbon Nanotubes-LiFePO_4_-Al Foam. Batteries.

[178] Ding C., Lu Z., Li S., Wang Z., Yu P., Ye S. (2023). Microstructures, Thermal and Mechanical Properties of Al–Si-CNT Composites for Thermal Management Applications. Mater. Chem. Phys..

[179] Ding C., Yu K., Nodooshan H.R.J., Ye S., Yu P. (2022). Effect of Powder Microstructure on the Thermal and Mechanical Properties of Hot Extruded Al-CNT Composite. J. Alloys Compd..

[180] Geng K., Li S., Yang Y.F., Misra R.D.K. (2020). 3D Printing of Al Matrix Composites through in Situ Impregnation of Carbon Nanotubes on Al Powder. Carbon.

[181] Li X.X., Zhang H.M., Mu X.N., Fan Q.B., Cheng X.W., Cui Z.S., Chang S. (2024). Achieving High Thermal Conductivity in CNTs@GF/Al Laminates via a Rapid and Simple Strategy. Vacuum.

[182] Zhou L., Liu K., Yuan T., Liu Z., Wang Q., Xiao B., Ma Z. (2022). Investigation into the Influence of CNTs Configuration on the Thermal Expansion Coefficient of CNT/Al Composites. J. Mater. Res. Technol..

[183] Chen B., Kondoh K. (2016). Sintering Behaviors of Carbon Nanotubes—Aluminum Composite Powders. Metals.

[184] Zhang Z.-C., Zhang G.-W., Hu Y.-L., Lv W.-Z., Yu H., Ren X.-Y. (2024). Al-Ti-C (CNTs) Master Alloys Improve Room Temperature and High-Temperature Mechanical Properties of ZL205A Alloy. Mater. Today Commun..

[185] Parveez B., Kittur M.I., Badruddin I.A., Kamangar S., Hussien M., Umarfarooq M.A. (2022). Scientific Advancements in Composite Materials for Aircraft Applications: A Review. Polymers.

[186] Guo M., Du J., Zhang Y. (2023). Effect of CNT Content and Size on the High-Temperature Particle-Erosion Resistance of Ablative Materials for Thermal Protection Systems. Compos. Sci. Technol..

[187] Nguyen-Tran H.-D., Hoang V.-T., Do V.-T., Chun D.-M., Yum Y.-J. (2018). Effect of Multiwalled Carbon Nanotubes on the Mechanical Properties of Carbon Fiber-Reinforced Polyamide-6/Polypropylene Composites for Lightweight Automotive Parts. Materials.

[188] Wu D., Zhao S., Huang T., He W., Zhou X., Wang G., Guo M., Luo X., Cao M., Yue Y. (2024). Self-Charging V2CTx/CNT-Based Zinc Ion Micro-Supercapacitor for Wearable Electronics. Chem. Eng. J..

[189] Benali H., Hartiti B., Lmai F., Batan A., Fadili S., Thevenin P. (2024). Enhancing the Efficiency of the Organic-Inorganic Hybrid Perovskite Cells Using Al-Doped ZnO as an Electron Transport Layer and CNTs as a Hole Transport Layer: An Experimental and Numerical Study. Optik.

